# "Times Are Changing": The Impact of HIV Diagnosis on Sub-Saharan Migrants’ Lives in France

**DOI:** 10.1371/journal.pone.0170226

**Published:** 2017-01-27

**Authors:** Anne Gosselin, Eva Lelièvre, Andrainolo Ravalihasy, Nathalie Lydié, France Lert, Annabel Desgrées du Loû, for the Parcours Study Group

**Affiliations:** 1 CEPED, UMR 196 Université Paris Descartes–IRD, 45 rue des Saints Pères, Paris, France; 2 INED (French Institute for Demographic Studies), 133 Bd Davout, Paris, France; 3 Santé Publique France—Site Carrefour Pleyel, 42 Bd de la Libération, Saint-Denis, France; 4 CESP, INSERM, Hôpital Paul Brousse, Villejuif, France; 5 IRD (Institut de Recherche pour le Développement), 44 Bd de Dunkerque, Marseille, France; Agencia de Salut Publica de Barcelona, SPAIN

## Abstract

**Background:**

Migrants account for 35% of HIV diagnoses in the European Union (ECDC/WHO 2014). Little is known about the impact of such a lifelong infection diagnosis on lives that are already disrupted by migration. In this paper, we assess the impact of HIV diagnosis on activity, union, well-being among African migrants living in France, the second group most affected by HIV after MSM. We compare it with the impact of the diagnosis of Hepatitis B, another lifelong infection affecting African migrants.

**Methods:**

We use the ANRS PARCOURS survey, a retrospective life-event survey led in 2012–2013 in 74 health structures in Paris greater area which collected 926 life histories of Sub-Saharan migrants living with HIV and 779 with Hepatitis B. We modelled the probability year by year since 18 years of age until data collection to lose one’s activity, to experience a conjugal break up and degradation of well-being and we estimated the impact of migration and of HIV and Hepatitis B diagnoses on these probabilities, after adjustment on other factors, thanks to discrete-time logistic regressions.

**Results:**

Migration entailed loss of activity and conjugal break up, though HIV diagnosis after migration did not statistically impact on these outcomes. Yet HIV diagnosis had a massive negative impact on well-being (aOR = 11.31 [4.64–27.56] for men and 5.75 [2.79–11.86] for women). This negative impact on well-being tended to diminish for persons diagnosed after 2004. The negative impact of HIV diagnosis on African migrants’ well-being seems to be attenuated in the last decade, which hints at a normalization of the subjective experience of HIV diagnosis.

## Introduction

Migrants living in Europe account for 35% of HIV incident diagnoses of HIV/AIDS [1] and persons coming from Sub-Saharan Africa represented 31% of new HIV diagnoses in France in 2013 [2]. HIV/AIDS may entail a biographical disruption in people’s lives [3] and impact many spheres, such as patients’ professional lives, intimate ties, social networks, sexuality, and more generally life expectations and well-being. This was especially the case at the beginning of the AIDS era, when an HIV-positive diagnosis was tantamount to a death sentence. Previous work has shown that HIV-infection negatively affected persons’ chances of accessing employment, particularly among women [4,5]. It was also shown that persons living with HIV (PLHIV) could experience negative reactions from their partners and spouse when they learned about their infection [6] as well as within the family circle [7]. However, the arrival of HIV can give access to a legal permit for health reasons for undocumented migrants and it was also shown in other population to be a moment of self-reconstruction [8]. Migrants arriving in France have already experienced a disruption of their lives because they have left their home countries. Indeed, the migration pathway often has its pitfalls, provokes long-lasting separations of families [9] and causes occupational downgrading [10,11]. What, then, is the impact of HIV diagnosis on lives that are already disrupted by migration?

With the progressive transformation of HIV/AIDS into a chronic illness, the social impact of HIV/AIDS may have changed. The debate about whether HIV/AIDS could be a ‘chronic disease like any other’ emerged at the arrival of effective treatments in 1996 [12]. Qualitative research suggests that the medicalization of HIV disease has contributed to the normalization process, however stigma is still challenging this normalization in Europe as elsewhere [13,14].

The objective of this paper is to assess the impact of HIV diagnosis on the lives of Sub-Saharan migrants living in France using data from a large life-event survey of people from sub-Saharan Africa living in France: the ANRS PARCOURS survey. We analyze the respective impacts of HIV diagnosis and migration, and by studying diagnoses before and after the arrival of ARTs, we wish to question the ‘normalization’ of life with HIV.

To test the possible transformation of the impact of HIV diagnosis, the PARCOURS data offer the unique opportunity to compare HIV diagnosis with the diagnosis of Hepatitis B, another chronic infectious disease that affects disproportionately migrants from Sub-Saharan Africa. Indeed, 5.25% of Sub-Saharan migrants in France have chronic hepatitis B vs. 0.65% in the general population [15]. HIV and Hepatitis B share some transmission routes (mother to child, sexual intercourse, blood transfusion), and both require lifelong treatments. Very few studies have dealt with the potential social consequences of chronic Hepatitis B, and they could be very different from the impact of an HIV diagnosis. On the one hand, chronic Hepatitis B is a disease potentially less devastating than HIV because of longer asymptomatic period and low proportion of people with severe complications. On the other hand, previous work suggests that in the French setting, PLHIV may benefit from better support than PLCHB (people living with chronic Hepatitis B) because PLHIV might be offered comprehensive social care [16]. Indeed, the early community mobilization to fight AIDS has led to community organization and support for people in need and to a comprehensive approach of care [17].

Thus, the respective impacts of these two diseases can be questioned: even if HIV/AIDS benefits from an exceptional mobilization in comparison to chronic Hepatitis B, its normalization as a ‘chronic disease like any other’ may be challenged by health complications, decreased self-esteem, stigmatization and discrimination as well as social hardships that PLHIV may experience [18,19].

In this paper, we assess the impact of HIV diagnosis on the lives of Sub-Saharan migrants living in France. We use data from the PARCOURS life-event biographical survey, which collected information on the life trajectories of Sub-Saharan migrants living with HIV or living with chronic Hepatitis B. We propose to look at this impact on the probabilities to lose one’s activity, to experience conjugal break up and a degradation of well-being across time. We analyze the respective impacts of HIV diagnosis and migration and we compare the impact of HIV diagnosis to the impact of diagnosis of chronic Hepatitis B.

## Material and Methods

### Population of interest

The ANRS PARCOURS study was conducted to analyze how health trajectories and social and migratory paths are interlaced for migrants from Sub-Saharan Africa living in France. This retrospective quantitative life-event survey was conducted from February 2012 to May 2013 in healthcare facilities in the greater Paris metropolitan area (Ile-de-France) among two groups of migrants born in Sub-Saharan Africa: one receiving HIV care, one with chronic hepatitis B (and not HIV infected). Patients were eligible if they were born in Sub-Saharan Africa, aged 18 to 59, and diagnosed at least 3 months earlier. HIV and Hepatitis B groups were randomly sampled and are representative of migrants followed for these pathologies in the Paris metropolitan area. All information was anonymously collected. The Advisory Committee on Data Collection in Health Research (CCTIRS) and the French Data Protection Authority (CNIL) both approved this study.

Physicians asked all eligible patients, except those with major cognitive or health impairments, to participate and collected their written consent. A trained interviewer administered a face-to-face standardized life-event history questionnaire to each participant. Information collected included socio-demographic characteristics; conditions of migration and life in France; relational, sexual, and reproductive history; and healthcare pathways. Each dimension of interest was documented year by year from birth until the time of data collection. The complete survey protocol is registered on Clinicaltrials.gov (NCT02566148) and available online (http://ceped.org/parcours/protocol-en.pdf), (See S1 File). The study’s recruitment and design have been extensively described elsewhere [20].

As we are interested in looking at what happens before and after the moment of diagnosis, and because we understand that social impacts can unfold for several years after diagnosis, we exclude people who have been diagnosed recently (in the five years preceding the survey). Being diagnosed as a child can eventually entail very different consequences, and in any case, occupational and legal status situations are hardly comparable between children and adults. For this reason, we exclude persons who were diagnosed or who migrated to France before 18 years of age. Only about 10% of the remaining sample had been diagnosed in their country of origin, the rest was diagnosed for HIV or Hepatitis B after arrival in France. Because the social and sanitary circumstances are very different in the country of origin and in France, the impact of diagnosis could differ as well. For this reason, we chose to exclude persons diagnosed before migration from this analysis. Eventually, about 20% of the remaining sample had migrated and be diagnosed the same year. As our objective was to distinguish the impact of the two events, we excluded this population from the study.

### Outcomes and variables of interest

To consider the impact of diagnosis on both living conditions and well-being, we looked at three outcomes for each year between 18 years of age and the date of data collection: being in activity, being in union and the level of perceived well-being.

Being in activity (yes/no for a given year) means having a job (regardless of the job being in the formal sector or not) or being a student during most of the year considered. Being in union (yes/no for a given year) is defined as being in a relationship that lasted at least a year, regardless of marital status or whether ego lives with his or her partner. Eventually, the persons were asked to indicate which periods in their life they considered as ‘good years’, ‘difficult years’ or ‘neither good nor bad years’. Well-being (yes/no for a given year) is then defined as a year mentioned as a ‘good year’ or a ‘neither good nor bad year’, a global indicator for well-being tested in other surveys [21].

To look at the impact of diagnosis (either HIV or chronic hepatitis B), we distinguished for each patient the period before diagnosis, the period just after diagnosis (year of the diagnosis and year after) and the period after. To look at the impact of migration, we distinguished for each patient the period before migration, the period just after migration (year of migration and year after) and the period after.

To account for dramatic changes in the prognosis of HIV infection thanks to advances in ART, we considered three different periods: before 1996 (the arrival of effective ART (Antiretroviral Therapy)), between 1996 and 2004 (when access to treatment existed in France but not in Sub-Saharan Africa) and after 2004, when the scale-up of treatments began in Sub-Saharan Africa, although important issues remain regarding access [22].

### Control variables

All the models are adjusted on socio-demographic characteristics of the interviewees, which can influence their migratory trajectory. These include region of origin (West Africa, Central Eastern and Southern Africa), education level (none or primary, secondary, superior), and reasons for migration (take a chance, join a family member, threatened in country of origin, study, medical reasons). Two time-dependent variables were included: age in four categories (18–24; 25–34; 35–44; 45–59) and to have a child birth in a given year (yes/no).

### Statistical analysis

We approached the measure of impact in two ways: first we looked at the proportions of persons in activity, union and well-being over time, and then we focused the analysis on the probability to experience an activity loss, conjugal break up and well-being degradation and the factors associated with these outcomes.

To compare the respective impacts of HIV diagnosis and migration, we described the distribution of the proportions of persons in activity, in union and their perceived well-being over time, in relation to migration and diagnosis, by group and sex using relative-time graphs. This method was first designed by Brou and colleagues [23] and was adapted to our dataset and analyses (See S2 File). These curves allow us to see how the proportions vary over time and what the respective impacts of migration and HIV diagnosis are. We then described with similar curves the respective impacts of migration and diagnosis of chronic Hepatitis B.

To observe whether the impact of HIV diagnosis has changed over time, we compared with a paired chi2-test the proportions of persons who were in activity, in union and their perceived well-being the year before diagnosis and the year after, stratified by period of HIV diagnosis (before 1996, 1996–2004 and after 2004) to account for the increasing availability of treatment. We performed the same analysis for chronic Hepatitis B diagnosis.

Secondly, we modeled the probability of losing activity (transition from activity or studies towards inactivity), of conjugal break ups and degradation of well-being (transition from ‘good years’ or ‘neither good nor bad years’ towards ill-being). The associations year by year (since 18 years of age) between transition towards inactivity and the situation regarding migration and diagnosis were analyzed with discrete-time logistic regression models for recurrent events, per sex and study group (HIV group and Hepatitis B group). Discrete-time logistic regressions allowed us to measure associations year by year between the outcomes and variables of interest [24]. Multivariate models were adjusted for time-independent variables (region of origin, education level, reasons for migration) and also for time-dependent variables (age, period, child birth). The same models were used to look at the factors associated with conjugal break-up and transition from well-being towards ill-being.

Data were weighted according to each individual’s probability of inclusion in the survey, and the weights applied to all percentages. All analyses were performed in Stata SE 12.1 [25].

## Results

### Study population

The characteristics of the population are given by study group and by sex (Table 1). In total, 1705 persons were included in the HIV and Hepatitis B groups in the PARCOURS survey (926 with HIV and 779 with chronic hepatitis B). A total of 601 had been diagnosed less than five years earlier, 131 were diagnosed or had migrated before 18 years of age, 118 had been diagnosed before migration, 204 were diagnosed the same year than migration and for 22 persons there were missing data. Ultimately, 629 persons were included in the study (371 HIV-positive, 258 with chronic Hepatitis B). The median age of migrants living with HIV was 48 years old for men and 43 years old for women; migrants living with chronic Hepatitis B were slightly younger. The overall population had arrived in France 13 to 20 years earlier (median). Whereas family reunification constitutes one of the main reasons why women came to France, men mostly came to “find a better life than at home”.

**Table 1:** Socio-demographic characteristics of the studied population at arrival in France and at time of survey.

	Men				Women			
	HIV (N = 142)		HepB (N = 192)		HIV (N = 229)		HepB (N = 66)	
	%	*n*	%	*n*	%	n	%	n
**Age at time of survey**								
Median (IQR)	48 (42;54)		44 (40;51)		43 (37;49)		40 (36;47)	
**Region of origin**								
West Africa	57	*78*	77	*148*	61	*134*	87	*54*
Central, Eastern and Southern Africa	43	*64*	23	*44*	39	*95*	13	*12*
**Education level at arrival in France**								
None, primary	28	*38*	37	*74*	28	*60*	27	*17*
Secondary	45	*65*	35	*66*	55	*133*	63	*42*
Superior	27	*39*	28	*52*	17	*36*	10	*7*
**Median year of arrival in France (IQR)**	1993(1987;2001)		1999(1990;2001)		1999(1992;2001)		2001 (1995;2002)	
**Median length of stay (IQR)**	20 (12;26)		15 (12;23)		14(12;21)		13 (11;18)	
**Median age of arrival in France (IQR)**	27 (24;33)		27 (24;32)		27 (23;32)		26 (23;31)	
**Reason for migration**								
Take a chance	46	*70*	56	*107*	38	*84*	30	*22*
Join a family member	8	*10*	7	*16*	38	*88*	51	*32*
Threatened in country of origin	23	*30*	13	*23*	10	*26*	6	*4*
Studies	22	*29*	23	*42*	12	*27*	6	*4*
Medical reasons	1	*3*	1	*4*	2	*4*	8	*4*
**Period of arrival**								
Before 1996	53	*78*	44	*79*	31	*80*	27	*19*
1996–2004	44	*61*	50	*99*	62	*131*	64	*41*
After 2004	3	*3*	7	*14*	7	*18*	9	*6*
**Median year of HIV or Hepatitis B diagnoses (IQR)**	2004 (1998;2006)		2004 (2001;2006)		2002 (1999;2005)		2003(2002;2005)	
**Median age at time of diagnosis (IQR)**	37 (31;42)		35(30;38)		32 (27;37)		32 (27;35)	
**Time spent between migration and diagnosis**								
Diagnosis one year after migration	19	*29*	16	*34*	25	*74*	32	*21*
Diagnosis from the 3d year in France and following	81	*113*	84	*158*	75	*155*	68	*45*
**Legal situation at time of survey**								
No permit or less than 1 year	15	*15*	21	*43*	9	*21*	12	*8*
Short-term permit	30	*45*	31	*57*	27	*67*	27	*15*
Long-term permit	55	*82*	48	*92*	65	*141*	61	*43*
**Activity at time of survey**								
No activity	19	*27*	16	*31*	28	*60*	31	*21*
Studies	2	*3*	2	*4*	3	*5*	5	*2*
Activity	79	*112*	83	*157*	70	*164*	64	*43*
**Couple situation at time of survey**								
In union	61	*88*	79	*153*	65	*149*	69	*45*
**Well-being situation at time of survey**								
Ill-being	26	*37*	16	*32*	21	*48*	20	*14*
Intermediary	26	*29*	16	*35*	20	*46*	16	*11*
Well-being	48	*76*	68	*125*	59	*135*	64	*41*

Scope: persons of HIV and Hepatitis B groups who have not yet migrated or being diagnosed at 18 years of age, who are observed at least five years after diagnosis, who were diagnosed in France at least one year after arrival

Source: ANRS Parcours Survey 2012–2013

### The effect of migration and diagnosis on activity, union and well-being

**Fig 1:**
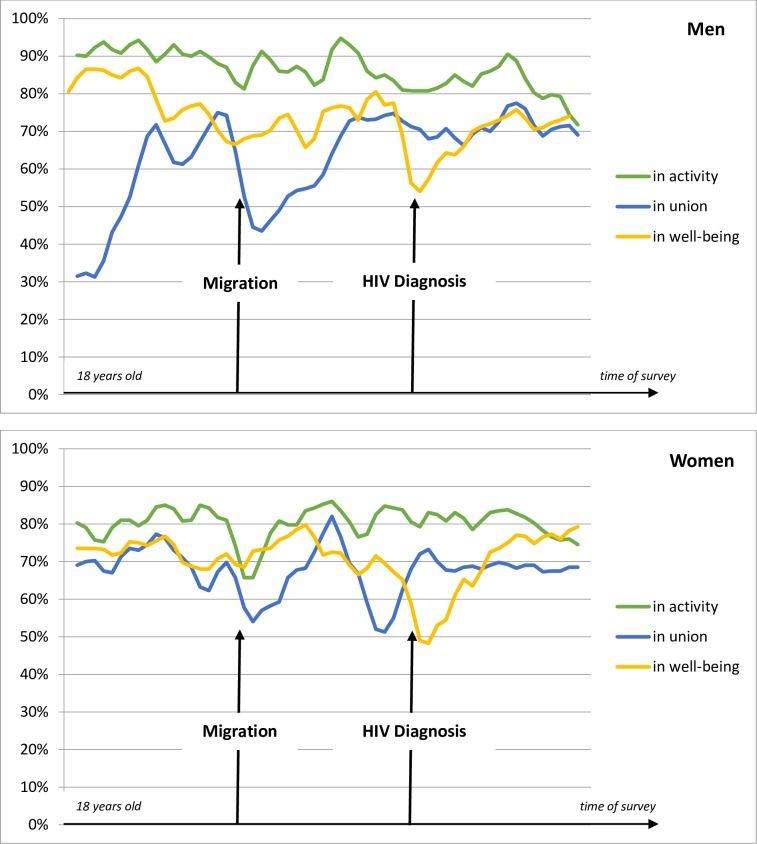
Persons living with HIV: proportion of men and women in activity, union and well-being in relation to migration and HIV diagnosis. Time-relative graphs Scope: men (N = 142) and women (N = 229) from HIV group who have not yet migrated or being diagnosed at 18 years of age, who are observed at least five years after diagnosis, who were diagnosed in France at least one year after arrival. Note: These curves show the proportion of persons in activity, in union and their perceived well-being in relation to two key moments: migration and diagnosis, for persons who were diagnosed after migration. For more details, please see S2 File and Brou et al. (2007). Source: ANRS Parcours Survey 2012–2013.

Migration entailed a sharp drop in the proportion of men in union among men of the HIV group (Fig 1). On the other hand, the level of activity goes down slightly but goes up again and stays at a relatively high level; the well-being does not seem to be affected by migration, at a population level. The HIV diagnosis is followed by a slight drop in activity and union rates and it provokes a sharp degradation of well-being.

**Fig 2:**
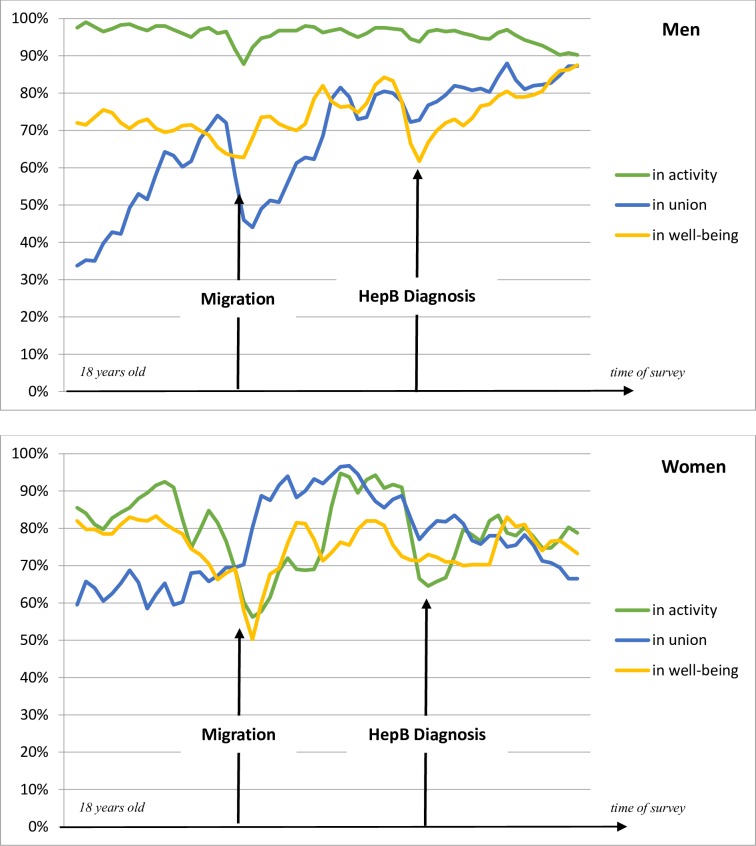
Persons living with chronic Hepatitis B: proportion of men and women in activity, union and well-being in relation to migration and chronic Hepatitis B diagnosis. Time-relative graphs Scope: men (N = 192) and women (N = 66) from Hepatitis B group who have not yet migrated or being diagnosed at 18 years of age, who are observed at least five years after diagnosis, who were diagnosed in France at least one year after arrival. Note: These curves show the proportion of persons in activity, in union and their perceived well-being in relation to two key moments: migration and diagnosis, for persons who were diagnosed after migration. For more details, please see S2 File and Brou et al. (2007). Source: ANRS Parcours Survey 2012–2013.

Among men from the Hepatitis B group, we observe the same changes at the moment of migration: there are less men in union, whereas activity and well-being are not affected; the proportion of men in well-being even increases progressively after migration (Fig 2). The Hepatitis B diagnosis is also followed by a drop in well-being among men but to a lesser extent than HIV diagnosis. Thus the curves look quite similar between HIV and Hepatitis groups among men, however the activity rate decreases progressively in the HIV group, which is not the case in the Hepatitis B group.

Among women from the HIV group, migration entails both a drop in union and activity levels (Fig 1). An important decrease of the proportion of women in union is visible before the HIV diagnosis. The HIV diagnosis itself does not seem to affect activity or union among women, but it provokes an important drop of the proportion of women in well-being.

Women from the Hepatitis B group are quite different from the HIV group, as they experience a marked decrease of activity and well-being levels at the time of migration, as well as an increase of union levels (Fig 2). At the moment of Hepatitis B diagnosis, the proportion of women in activity decreases. Well-being does not seem affected by Hepatitis B diagnosis when the effect on well-being was very strong among women from the HIV group.

Thus HIV diagnosis as such does not seem to affect activity nor union, at a population level, but it is followed by a degradation of women’ well-being.

**Fig 3:**
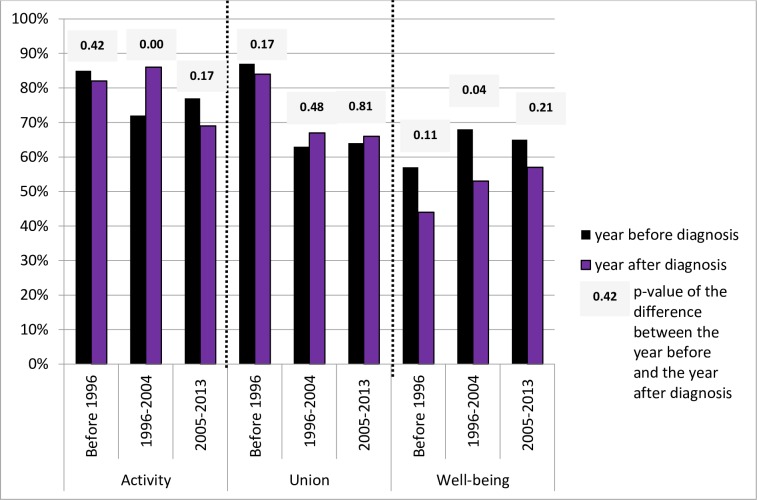
Persons living with HIV: proportion of persons in activity, union and well-being the year before and the year after HIV diagnosis, according to the period of diagnosis (N = 371). Scope: men (N = 142) and women (N = 229) from HIV group who have not yet migrated or being diagnosed at 18 years of age, who are observed at least five years after diagnosis and who were diagnosed in France at least one year after arrival. Source: ANRS Parcours Survey 2012–2013.

When comparing the proportions of persons in activity, in union and with perceived well-being just before and just after HIV diagnosis, according to the period of diagnosis, it is noteworthy that before the arrival of antiretroviral treatments in 1996, HIV diagnosis entailed a decrease of well-being levels, without any effect on activity nor union levels (Fig 3). During the early years of effective ART (1996–2004), union levels remained unaffected by HIV/diagnosis however, diagnosed persons were even more often in activity after diagnosis as the access to treatment was possible in France at that time (p<0.00). Still, diagnosis was followed by a major decrease in perceived well-being (p<0.04). However, after 2004, the decrease in the well-being rate is no longer statistically significant (p = 0.21), hinting at an improvement in subjective experience of HIV diagnosis. When comparing the proportions of persons in activity, in union and with perceived well-being just before and just after chronic Hepatitis B diagnosis, there was no significant difference according to the period of diagnosis (see S1 Fig).

The multivariate analyses (Tables 2–4) allow us to measure for each indicator (activity, union, well-being) the associations with migration and diagnoses, everything being equal.

**Table 2:** Factors associated with transition towards inactivity year by year since 18 years of age until data collection.

	Men		Women	
	HIV	Hepatitis B	HIV	Hepatitis B
	3698 years at risk	4740 years at risk	4534 years at risk	1207 years at risk
	aOR [CI95%]	aOR [CI95%]	aOR [CI95%]	aOR [CI95%]
**Migrationǂ**				
Has not migrated yet	1.00 [1.00,1.00]	1.00 [1.00,1.00]	1.00 [1.00,1.00]	1.00 [1.00,1.00]
Year of migration and following year	8.02***[3.55,18.11]	18.82***[8.20,43.16]	8.73***[5.09,14.99]	6.15***[2.17,17.40]
3d year in France and beyond	1.82 [0.82,4.03]	1.35 [0.40,4.55]	1.43 [0.72,2.80]	1.00 [0.42,2.40]
**Diagnosisǂ**				
Has not been diagnosed yet	1.00 [1.00,1.00]	1.00 [1.00,1.00]	1.00 [1.00,1.00]	1.00 [1.00,1.00]
Year of diagnosis and following year	1.20 [0.49,2.91]	0.85 [0.42,1.72]	0.78 [0.36,1.69]	0.52 [0.16,1.71]
3d year after diagnosis and beyond	0.89 [0.38,2.12]	1.11 [0.48,2.59]	0.77 [0.36,1.66]	0.53 [0.19,1.44]
**Period ǂ**				
Before 1996	1.00 [1.00,1.00]	1.00 [1.00,1.00]	1.00 [1.00,1.00]	1.00 [1.00,1.00]
1996–2004	1.39 [0.84,2.28]	2.40** [1.26,4.59]	1.77* [1.14,2.74]	1.70 [0.64,4.56]
After 2004	1.04 [0.55,1.96]	3.45** [1.42,8.38]	2.78** [1.46,5.29]	3.69 [0.87,15.67]
**Region of origin**				
West Africa	1.00 [1.00,1.00]	1.00 [1.00,1.00]	1.00 [1.00,1.00]	1.00 [1.00,1.00]
Central, Eastern and Southern Africa	2.07** [1.25,3.44]	0.64 [0.33,1.27]	1.51* [1.04,2.20]	0.72 [0.32,1.62]
**Education level at arrival in France**				
None, primary	1.00 [1.00,1.00]	1.00 [1.00,1.00]	1.00 [1.00,1.00]	1.00 [1.00,1.00]
Secondary	1.21 [0.70,2.09]	1.38 [0.78,2.46]	1.28 [0.78,2.09]	2.65* [1.25,5.63]
Superior	1.00 [0.50,1.98]	2.27 [0.97,5.31]	1.06 [0.56,1.99]	2.95* [1.13,7.66]
**Reason for coming to France**				
Work, take a chance	1.00 [1.00,1.00]	1.00 [1.00,1.00]	1.00 [1.00,1.00]	1.00 [1.00,1.00]
Family	0.68 [0.18,2.54]	0.96 [0.39,2.40]	1.24 [0.83,1.86]	1.63 [0.83,3.21]
Threatened in country of origin	1.06 [0.66,1.70]	1.01 [0.50,2.03]	1.27 [0.79,2.03]	1.39 [0.19,10.37]
Studies	1.03 [0.53,2.01]	0.52 [0.23,1.20]	0.72 [0.37,1.41]	0.22 [0.03,1.43]
Medical reasons	0.30 [0.03,2.71]	0.86 [0.15,5.02]	1.24 [0.61,2.54]	0.98 [0.40,2.43]
**Age in categoriesǂ**				
19–24	0.68 [0.26,1.77]	0.82 [0.36,1.90]	1.80 [0.89,3.63]	0.39 [0.11,1.43]
25–34	0.90 [0.46,1.76]	1.19 [0.63,2.26]	1.15 [0.71,1.85]	0.59 [0.27,1.27]
35–44	1.00 [1.00,1.00]	1.00 [1.00,1.00]	1.00 [1.00,1.00]	1.00 [1.00,1.00]
45–59	2.13* [1.13,4.03]	3.24** [1.44,7.29]	1.33 [0.78,2.26]	0.11** [0.02,0.57]
**Child birthǂ**	1.31 [0.69,2.49]	0.54 [0.23,1.30]	2.62*** [1.59,4.32]	2.62 [0.99,6.94]
**Timeǂ**	0.98 [0.95,1.00]	0.94** [0.91,0.98]	1.00 [0.97,1.03]	0.97 [0.91,1.03]

Discrete-time logistic models where v**ǂ** = time-dependent variable

*p<0,05

**: p<0,01

*** p <0,0001

Scope: persons of HIV and Hepatitis B groups who have not yet migrated or being diagnosed at 18 years of age, who are observed at least five years after diagnosis and who where diagnosed the year of migration of afterwards

HIV group: N = 142men and 228women (1 person never at risk); HepB group: N = 192men and 66women

Source: ANRS Parcours Survey 2012–2013

**Table 3:** Factors associated with conjugal break up year by year since 18 years of age until data collection.

	Men		Women	
	HIV	Hepatitis B	HIV	Hepatitis B
	2573 years at risk	3154 years at risk	3875 years at risk	1182 years at risk
	aOR [CI95%]	aOR [CI95%]	aOR [CI95%]	aOR [CI95%]
**Migration***ǂ*				
Has not migrated yet	1.00 [1.00,1.00]	1.00 [1.00,1.00]	1.00 [1.00,1.00]	1.00 [1.00,1.00]
Year of migration and following year	3.70*** [1.83,7.48]	3.29***[1.64,6.58]	3.00*** [1.68,5.35]	2.01 [0.64,6.31]
3d year in France and beyond	0.98 [0.52,1.84]	0.81 [0.41,1.62]	0.91 [0.53,1.56]	0.62 [0.19,2.01]
**Diagnosis***ǂ*				
Has not been diagnosed yet	1.00 [1.00,1.00]	1.00 [1.00,1.00]	1.00 [1.00,1.00]	1.00 [1.00,1.00]
Year of diagnosis and following year	1.00 [0.44,2.26]	0.36 [0.13,1.02]	1.23 [0.72,2.11]	1.14 [0.28,4.73]
3d year after diagnosis and beyond	1.10 [0.59,2.05]	0.65 [0.33,1.29]	0.83 [0.45,1.53]	0.41 [0.09,1.80]
**Period** *ǂ*				
Before 1996	1.00 [1.00,1.00]	1.00 [1.00,1.00]	1.00 [1.00,1.00]	1.00 [1.00,1.00]
1996–2004	1.44 [0.78,2.69]	1.72 [1.00,2.98]	1.45 [0.99,2.13]	3.06* [1.09,8.62]
After 2004	1.55 [0.72,3.34]	2.69* [1.18,6.12]	0.92 [0.51,1.68]	4.68* [1.28,17.15]
**Region of origin**				
West Africa	1.00 [1.00,1.00]	1.00 [1.00,1.00]	1.00 [1.00,1.00]	1.00 [1.00,1.00]
Central, Eastern and Southern Africa	1.54* [1.01,2.34]	1.17 [0.72,1.92]	1.00 [0.69,1.44]	0.44* [0.21,0.91]
**Education level at arrival in France**				
None, primary	1.00 [1.00,1.00]	1.00 [1.00,1.00]	1.00 [1.00,1.00]	1.00 [1.00,1.00]
Secondary	0.87 [0.51,1.49]	1.31 [0.52,3.26]	0.70 [0.47,1.04]	1.08 [0.53,2.19]
Superior	0.41* [0.19,0.88]	0.78 [0.30,2.03]	0.69 [0.39,1.22]	0.63 [0.26,1.55]
**Reason for coming to France**				
Work, take a chance	1.00 [1.00,1.00]	1.00 [1.00,1.00]	1.00 [1.00,1.00]	1.00 [1.00,1.00]
Family	1.55 [0.69,3.45]	0.86 [0.41,1.81]	0.83 [0.58,1.18]	0.43* [0.20,0.92]
Threatened in country of origin	1.13 [0.68,1.88]	1.37 [0.33,5.64]	1.14 [0.68,1.90]	0.48 [0.12,1.96]
Studies	1.79 [0.94,3.42]	1.55 [0.79,3.08]	1.10 [0.62,1.95]	2.55* [1.25,5.19]
Medical reasons	2.27** [1.23,4.19]	1.76 [0.52,5.95]	0.70 [0.24,2.06]	1.10 [0.27,4.47]
**Age in categories***ǂ*				
19–24	1.44 [0.68,3.04]	2.52 [0.98,6.48]	0.86 [0.46,1.60]	0.65 [0.19,2.25]
25–34	1.17 [0.64,2.15]	1.50 [0.82,2.75]	0.92 [0.54,1.54]	1.04 [0.44,2.48]
35–44	1.00 [1.00,1.00]	1.00 [1.00,1.00]	1.00 [1.00,1.00]	1.00 [1.00,1.00]
45–59	0.68 [0.33,1.42]	0.77 [0.33,1.84]	1.60 [0.80,3.21]	1.29 [0.42,3.94]
**Child birth***ǂ*	0.23*[0.07,0.71]	0.24* [0.08,0.73]	0.23**[0.09,0.56]	0.17* [0.04,0.76]
**Time***ǂ*	0.97 [0.94,1.01]	0.98 [0.94,1.04]	0.97*[0.95,1.00]	1.02 [0.97,1.07]

Discrete-time logistic models where vǂ = time-dependent variable

*p<0,05

**: p<0,01

*** p <0,0001

Scope: persons of HIV and Hepatitis B groups who have not yet migrated or being diagnosed at 18 years of age, who are observed at least five years after diagnosis and who where diagnosed the year of migration of afterwards

HIV group: N = 138 men and 224 women (9 persons never at risk); HepB group: N = 186 men and 65 women (7 persons never at risk)

Source: ANRS Parcours Survey 2012–2013

**Table 4:** Factors associated with transition towards ill-being year by year since 18 years of age until data collection.

	Men		Women	
	HIV	Hepatitis B	HIV	Hepatitis B
	3028 years at risk	3637 years at risk	3946 years at risk	1206 years at risk
	aOR [CI95%]	aOR [CI95%]	aOR [CI95%]	aOR [CI95%]
**Migration***ǂ*				
Has not migrated yet	1.00 [1.00,1.00]	1.00 [1.00,1.00]	1.00 [1.00,1.00]	1.00 [1.00,1.00]
Year of migration and following year	7.06***[3.51,14.21]	9.77***[5.13,18.60]	14.95*** [7.84,28.51]	7.75**[2.28,26.40]
3d year in France and beyond	0.53 [0.26,1.09]	0.93 [0.50,1.75]	3.98*** [1.89,8.39]	0.71 [0.16,3.25]
**Diagnosis***ǂ*				
Has not been diagnosed yet	1.00 [1.00,1.00]	1.00 [1.00,1.00]	1.00 [1.00,1.00]	1.00 [1.00,1.00]
Year of diagnosis and following year	11.31***[4.64,27.56]	2.25 [0.99,5.13]	5.75*** [2.79,11.86]	1.17 [0.30,4.54]
3d year after diagnosis and beyond	0.65 [0.25,1.67]	0.43 [0.17,1.07]	0.28** [0.13,0.64]	1.48 [0.52,4.23]
**Period** *ǂ*				
Before 1996	1.00 [1.00,1.00]	1.00 [1.00,1.00]	1.00 [1.00,1.00]	1.00 [1.00,1.00]
1996–2004	1.04 [0.62,1.76]	1.84** [1.17,2.90]	1.14 [0.68,1.90]	1.59 [0.61,4.15]
After 2004	1.42 [0.58,3.48]	1.86 [0.79,4.39]	1.58 [0.64,3.89]	2.87 [0.68,12.07]
**Region of origin**				
West Africa	1.00 [1.00,1.00]	1.00 [1.00,1.00]	1.00 [1.00,1.00]	1.00 [1.00,1.00]
Central, Eastern and Southern Africa	1.02 [0.57,1.82]	1.10 [0.75,1.61]	0.91 [0.58,1.42]	1.04 [0.33,3.28]
**Education level at arrival in France**				
None, primary	1.00 [1.00,1.00]	1.00 [1.00,1.00]	1.00 [1.00,1.00]	1.00 [1.00,1.00]
Secondary	0.83 [0.39,1.77]	1.23 [0.72,2.10]	0.81 [0.49,1.34]	1.07 [0.49,2.33]
Superior	0.96 [0.36,2.54]	1.80 [0.88,3.68]	0.80 [0.40,1.60]	0.93 [0.17,5.06]
**Reason for coming to France**				
Work, take a chance	1.00 [1.00,1.00]	1.00 [1.00,1.00]	1.00 [1.00,1.00]	1.00 [1.00,1.00]
Family	1.30 [0.75,2.26]	0.58 [0.27,1.24]	0.79 [0.47,1.33]	0.37* [0.17,0.84]
Threatened in country of origin	2.28** [1.23,4.23]	1.50 [0.96,2.35]	1.36 [0.72,2.56]	1.54 [0.56,4.19]
Studies	0.90 [0.35,2.28]	0.93 [0.54,1.60]	0.70 [0.34,1.46]	0.95 [0.20,4.61]
Medical reasons	0.93 [0.48,1.81]	0.38 [0.03,4.04]	0.32 [0.04,2.70]	0.28 [0.04,2.10]
**Age in categories***ǂ*				
19–24	1.41 [0.48,4.16]	1.91 [0.88,4.12]	3.67*** [1.77,7.58]	2.79 [0.59,13.19]
25–34	2.40* [1.15,5.00]	1.28 [0.73,2.23]	2.37** [1.41,3.98]	3.58* [1.13,11.34]
35–44	1.00 [1.00,1.00]	1.00 [1.00,1.00]	1.00 [1.00,1.00]	1.00 [1.00,1.00]
45–59	0.60 [0.21,1.72]	0.86 [0.35,2.08]	0.56 [0.19,1.68]	0.87 [0.15,5.22]
**Child birth***ǂ*	0.91 [0.47,1.78]	1.50 [0.78,2.90]	1.25 [0.61,2.54]	1.06 [0.33,3.39]
**Time***ǂ*	1.04 [1.00,1.09]	1.05* [1.01,1.08]	1.06** [1.02,1.10]	0.98 [0.93,1.04]

Discrete-time logistic models where vǂ = time-dependent variable

*p<0,05

**: p<0,01

*** p <0,0001

Scope: persons of HIV and Hepatitis B groups who have not yet migrated or being diagnosed at 18 years of age, who are observed at least five years after diagnosis and who where diagnosed the year of migration of afterwards.

HIV group: N = 139 men and 222 women (10 persons never at risk); HepB group: N = 190 men and 65 women (persons never at risk)

Source: ANRS Parcours Survey 2012–2013

Migration is associated with a loss of activity for both men and women (Table 2): in both groups, the period after arrival in France is associated with an activity loss (aOR = 8.02 [3.55–18.11] and 18.82 [8.20–43.16] for men, 8.73 [5.09–14.99] and 6.15 [2.17–17.40] for women, in HIV and Hepatitis B groups, respectively). There is no statistical association between diagnosis and loss of activity. Among Hepatitis B men and HIV-positive women, this loss of activity was more frequent in the recent period (>2004) compared to before 1996.

The multivariate analysis also confirms the association between migration and union status, the period after arrival in France being associated with an increase in conjugal break up (aOR = 3.70 [1.83–7.48] for men and 3.00 [1.68–5.35] for women in the HIV group, for instance) (Table 3). On the contrary, there is no association between HIV or Hepatitis B diagnoses and conjugal break up. The probability of conjugal break up among Hepatitis B men increased after 2004 in comparison to the period before 1996 (aOR = 2.69 [1.18–6.12]) and this seems to be a tendency for several subgroups.

The probability of well-being degradation increased in the years following migration, in both groups and in both men (aOR = 7.06 [3.51–14.21] and 9.77 [5.13–18.60] in HIV and Hepatitis B groups, respectively) and women (aOR = 14.95[7.84–28.51] and 7.75 [2.28–26.40] in HIV and Hepatitis B groups, respectively) (Table 4). After HIV diagnosis, the probability of well-being degradation increased strongly (aOR = 11.31[4.64–27.56] for men and OR = 5.75[2.79–11.86] for women) whereas it was not the case after Hepatitis B diagnosis (association not significant). There was no statistical association between the period and the probability to experience a degradation of well-being.

## Discussion

In this study, we aimed at assessing the impact of HIV diagnosis on activity loss, conjugal break up (objective experience) and also on the degradation of well-being (subjective experience). The results show that HIV diagnosis does not impact on activity nor on union, at a population level, but it has a strong negative impact on well-being which still exists, even though it tends to diminish after the generalization of effective ARTs. In comparison, Hepatitis B diagnosis did not have any significant impact of the three indicators aforementioned, at a population level. These results suggest that HIV diagnosis remains a traumatic experience in the Sub-Saharan migrant population living in France, even if it does not have significant impact in terms of activity or partner loss. These findings also bring new elements on the respective influence of migration and HIV diagnosis on the difficulties encountered by African migrants living with HIV, and on the so-called “normalization” of HIV.

### Respective impacts of migration and diagnosis

Migration provokes important disruptions in terms of activity and union. Then, when arriving in France, migrants are often without a stable partner and without activity. This also corresponds to a long period of settlement where migrants do not have access to basic elements of security [26]. Yet HIV diagnosis often occurs during these first years post-migration: 19% of men and 25% of women discover their HIV infection one year after their arrival in France. Although the rapidity of diagnosis after arrival is an asset from the public health point of view, it means that migrants are diagnosed at a time when they have experienced several disruptions in their jobs and in their families. The diagnosis of Hepatitis B also often occurs in the first years in France (Table 1). This means that diagnoses occur frequently in this difficult period after migration and the specific social difficulties should be taken into account in migrants’ healthcare at time of diagnosis.

The absence of significant impact of HIV diagnosis on conjugal break-up calls into question the idea that HIV-related stigmatization could cause conjugal separations. This confirm previous findings in Côte d’Ivoire where it has been noted that conjugal break ups among HIV-positive women was not due to a negative reaction from their partner but rather to HIV positive women’ willingness to put an end to the weakest relationships when they had to cope with the HIV infection [27].

If HIV diagnosis does not have a major impact on union and on activity among Sub-Saharan migrants compared to migration,his diagnosis provokes a dramatic decrease in persons’ well-being, which seems to be attenuated in the last decade. This impressive degradation of well-being at the time of HIV diagnosis reflects the extent to which HIV/AIDS remains an upheaval in people’s lives in the sense that it brings about questions on their partnerships, their ability to have children, but also provokes lifelong treatments. Furthermore, this disease is still associated with negative representations. Previous studies have shown that patients’ discourses are haunted by previous and contradictory experiences with the virus, as is undoubtedly the case for persons who have witnessed family and friends die of HIV-related illnesses [19]. It is noteworthy that well-being seems to come back after the moment of diagnosis itself, and in particular among women, reflecting not only the positive impact of access to treatments on many aspects of life [28,29] but also the effect of accessing comprehensive social care through hospital services and charities [16,30].

### Questioning the normalization of HIV

For people diagnosed for HIV after 2004, the shock is attenuated, which could be explained by the access to effective treatment and the subsequent assimilation of HIV to a chronic disease ‘like another’. Our findings suggest a possible normalization of HIV/AIDS among Sub-Saharan migrants in France.

However, although the comparison between raw proportions of persons in ill-being before and after diagnosis according to the period seemed to indicate that the shock attenuated in recent years, this effect of the period is not statistically significant in multivariate models. Possibly, the subjective experience of HIV diagnosis remains a very distressful experience despite the access to effective treatment, with stigmatization and health-related uncertainty as background concerns [31]. However, another possibility is that our data is not the best to appreciate this change, because of a bias that we could call “perception bias”. Indeed, it is expected that someone interviewed about a recent diagnosis would be more likely to underline how difficult it is than someone who was diagnosed a long time ago and who has had the time to take some distance about this particular moment. Then, our data are appropriate to measure the degradation of well-being after HIV diagnosis, but it may be limited to evaluate the change of this subjective experience across time.

### The degradation of economic and social situation

When looking at all Sub-Saharan migrants, independently of HIV, the general situation they have to face in France has worsened over time. Both the hardening of migratory laws [32] and the economic crisis made it very difficult for migrants to secure employment and resident permit in the last decade [26]. The role of the economic crisis in the degradation of the employment situation for PLHIV was already noted [33], and our study confirms the recent degradation of the employment situation for migrants living with HIV. Indeed, all the Odds ratios for the recent period (after 2004) show a tendency to a higher risk of activity loss (even if they are statistically significant for two subgroups). Previous studies have shown how chronically-ill people can be excluded from labor market in time of economic crisis [34,35].

Additionally, migrants living with HIV face a double difficulty at the legal level, resulting from the European legislative restrictions on resident permits [32]. Furthermore, migrants living with HIV must face a specific discrimination towards persons holding a permit for healthcare reasons. Although they are entitled to the resident card, their access to a longer term permit is hindered by the fact that they are holding a permit for healthcare reasons, as suggested in previous study led in French Overseas Territories [36].

### Limitations and strengths

Our study has some limitations. First, we used synthetic indicators for activity and union (such as to be in union or not, to be in activity or not) and they cannot take into account changes in activity or partnership, thus we could not take into account the fact that work conditions could deteriorate after diagnosis for example. Also, the impact of HIV diagnosis on union status could possibly be the difficulty to find a new partner (celibacy) rather than losing a partner. Further research should investigate whether persons living with HIV take longer to find a partner because of their HIV status. Secondly, as the impact assessment as such is a methodological challenge, it may be especially difficult to assess impact retrospectively. Potential biases include the reconstruction of coherent meaning by the interviewees [37,38]. However, the life-event method of data collection facilitates recall of events and their ordering in time and it substantially reduces this bias [39,40]. The indicator for perceived well-being as such was tested and validated in other settings [21], however the retrospective data collection of perceived well-being may suffer from a perception bias, as seen above.

Also, since we reduced our sample to persons diagnosed at least a year after migration to France, our statistical sample was somehow reduced. However, all the analyses were performed including persons who experienced migration and diagnosis in the same year, and the results were similar.

Finally, it would have been interesting to take into account in our analyses a measure of persons’ health conditions at time of diagnosis, but it was not available in the survey.

The strength of the study, however, is to provide a global view of the social consequences of HIV/AIDS. Furthermore, the biographical approach allowed us to quantitatively measure individual situations at critical moments such as migration and diagnosis, thus enabling us to compare the impact of two diseases (HIV and Hepatitis B) and also to compare it between two key moments of persons’ life paths (migration and diagnosis).

The study was led in Paris area, but 60% of Sub-Saharan migrants in France live in this region. By comparing HIV/AIDS with chronic Hepatitis B, we confirm the specificity of HIV in terms of its degradation of well-being. However, we also show that the major disruption to social life is caused by migration, with the impact of migration appearing much more determinant in activity and union than the impact of HIV diagnosis.

## Conclusion

Migration entails great consequences for people’s lives. Public policy aiming at improving life with HIV should thus specifically address the difficult situation faced by Sub-Saharan migrants when they arrive in France. HIV diagnosis can trigger a degradation of well-being, but times are changing: with the generalization of effective ARTs, HIV diagnosis is less frightening that it used to be. Nevertheless, even if the situation is improving, hardship around diagnosis is still observed and further research is needed to understand why stigmatization remains important in the era of generalized antiretroviral therapy and how this can interact with persons’ well-being. On the other hand, migrants have to face the recent degradation of economic and social situation that has consequences on their lives. At a time when migrants are enduring renewed forms of fear and rejection in European societies, our results plead for appropriate social support at the critical moment of diagnosis which closely follows migration and constitutes a moment of important disruption.

## Supporting Information

S1 File ANRS Parcours survey Protocol in English. (PDF)

S2 File Construction of curves of Fig 1 and Fig 2. (DOCX)

S3 File STROBE Statement—Checklist of items that should be included in reports of *cross-sectional studies*, applied to the present study. (DOCX)

S1 Fig Proportion of persons in activity, union and well-being the year before and the year after chronic Hepatitis B diagnosis, according to the period of diagnosis, among persons diagnosed after arrival in France (N = 258). (DOCX)
