# Supplementary material for: "Times Are Changing": The Impact of HIV Diagnosis on Sub-Saharan Migrants’ Lives in France
Source: PLoS One. 2017 Jan 27;12(1):e0170226. doi: 10.1371/journal.pone.0170226 (PMC5271323; doi:10.1371/journal.pone.0170226)
Supplement: S1 File — (PDF) [file pone.0170226.s001.pdf]

ANRS-PARCOURS Study

« Life Course, HIV and Hepatitis B among African migrants living in Ile-de-France »

PROTOCOL

The *Parcours* study is conducted by a research team including researchers from IRD-Université Paris Descartes, INSERM and INPES. The *Parcours* study is funded by the National Research Agency against AIDS and Viral Hepatitis (ANRS), with the support of the General Directorate of Health, and is supported by associations of migrants and of HIV and hepatitis B patients. Data collection was conducted by ClinSearch (Clinical Research Organisation) and Ipsos (Survey Institute).

Brief summary :

Populations from Sub-Saharan Africa represent one of the most dynamic immigration flows in France and are among the most exposed to HIV infection and hepatitis B. Adjusting public health strategies to the specific needs this population requires improved knowledge on the processes that increase vulnerability to these diseases or otherwise contribute to the optimal benefit of prevention and care.

The objective of the *Parcours* study is to understand, among sub-Saharan African migrants, how social and individual factors combine in the course of migration and settlement in France, and increase or decrease the risk of infection, improve or reduce access to prevention and care, optimize or restrict the effectiveness of care for both HIV and hepatitis B diseases.

The research was conducted in Ile-de-France, where 60% of sub-Saharan African migrants reside. It consists in a cross-sectional observational survey, using a life-event history approach that reproduces the sequence of different life and health events, and contributes to explain the present situation (type of disease management, patient's quality of life) in light of all the elements of the past trajectory (administrative, familial, socioeconomic, professionals) and of the patient's future projects.

A representative survey was conducted between February 2012 and May 2013 in health care facilities in Ile-de-France, among three groups of migrants from Sub-Saharan Africa: a group living with HIV, a group living with chronic hepatitis B and a group who has neither of these pathologies. For each group, stratified random sampling was used. An exhaustive list of health care facilities was compiled and these were stratified according to their number of sub-Saharan migrants. In the end, the survey was conducted in 24 hospital services providing HIV care, 20 health care facilities providing hepatitis B care, and 30 primary health care facilities.

During the survey, were eligible all patients attending these health care facilities, born in a Sub-Saharan African country and with Sub-Saharan African citizenship at birth, aged 18 to 59 years, with an HIV diagnosis (HIV group) or chronic hepatitis B diagnosis (hepatitis B group) more than three months prior or not diagnosed with HIV or chronic Hepatitis B (Primary Health Care group). Health care professionals offered study participation to all eligible patients and collected their signed consent when they agreed to participate.

A total of 1,829 eligible patients in HIV services, 1,169 in hepatitis B facilities and 1,184 in primary health care facilities were identified, of which respectively 141, 25 and 124 were not offered study participation by their doctor, for health reasons mainly. Among the patients offered participation, 926 HIV-infected patients, 779 patients infected by hepatitis B, and 763 patients without these two diseases agreed to participate in the study. The characteristics of participants and non-participants were largely similar across the three types of facilities.

In order to take into account the sample design and non-participation, data was weighted according to each individual's probability of inclusion in the survey (i.e. considering the probability of inclusion in the sample for each health care facility, the number of half-days of weekly consultations in each facility included and the individual study participation per half-day of included consultations).
For all participants, detailed information on socio-demographic characteristics; migration and life conditions in France; social, sexual and reproductive life history; and screening and care history were collected using a life-event history questionnaire administered face-to-face by a specialized interviewer. Health care professionals documented clinical information from the medical records. Data was collected anonymously.

Each participant received a 15€ voucher compensation. The study was approved by the Advisory Committee for Data Processing in Health Research (CCTIRS) and by the National Commission on Informatics and Liberties (CNIL).

Detailed description:

The *Parcours* study is a cross-sectional observational study, using a life-event history approach, conducted within health care facilities in Ile-de-France. The survey took place between 30th January 2012 and 31st May 2013, among three representative groups of migrant populations from Sub-Saharan Africa living in Ile-de-France: people living with HIV/AIDS (HIV group), people with chronic hepatitis B (hepatitis B group), and people who have neither of these conditions and consult within primary health care facilities in Ile-de-France (reference group).

1. Sampling :

HIV group:

The random sample of HIV-infected individuals was created from the sampling frame of the ANRS-VESPA2 nationally representative survey among people infected with HIV/AIDS in France[1]. Of the 41 hospitals following HIV patients with in the Paris region, we selected the 37 services where at least 100 patients from sub-Saharan Africa were followed-up. Within this sampling frame, hospital services were selected by stratified randomisation. The 37 hospital were divided into five strata according to the number of Sub-Saharan patients in care: stratum 1 for 100 to 199 patients, stratum 2 for 200 to 299 patients, stratum 3 for 300 to 349 patients, stratum 4 for 350 to 600 patients and stratum 5 for 600 patients or above. A total of 27 services were sampled and 24 agreed to participate in the study (i.e. a participation rate of 89%). The sampling rate was 0,333 in stratum 1, 0.5 in stratum 2, 0.375 in stratum 3, 0.625 in stratum 4 and 1 for stratum 5. Based on the estimation of the required sample size (set to 1000 people), the sampling rate in each stratum was determined according to the weight of the number of patients in care in each stratum within the total population in the 5 strata.

Hepatitis B group:

The random sample of individuals diagnosed with chronic hepatitis B was created using a sampling frame of all the structures in Ile-de-France with sub-Saharan African patients receiving chronic hepatitis B care. This sampling frame was built during a phone pre-survey in 2011 conducted among the health hubs and networks as well as the hospitals in Ile-de-France, which provided information on the average number of sub-Saharan patients in hepatitis B care seen during a half-day in each service. All services reporting at least three sub-Saharan African patients daily were selected for the study. The survey was conducted among 20 of 26 such health care facilities (i.e. a facility participation rate of 77%) including: 7 hospital specialised in hepatology, 10 of the 16 other hospital services (not

[1] Dray-Spira R, Spire B, Lert F, et le groupe Vespa2. Méthodologie générale de l’enquête ANRS-Vespa2. Bull Epidémiol Hebd. 2013; (26-27):321-4.

specialised in hepatology – 6 refused to participate), the 2 "ville-hopital" health networks providing hepatitis care and the health centre of the Medical Committee for Exiles (Comede). In order to build a sample reflecting the contribution of different types of chronic hepatitis B care facilities in Ile-de-France, the number of individuals to include in each facility was determined according to its weight within the total population of patients from sub-Saharan Africa followed for chronic hepatitis B in Ile-de-France.

Primary care group:

The random sample of people without HIV or hepatitis B and consulting primary health care facilities was created using a sampling frame including the primary health care facilities of the National Federation of Health Centres (FNCS) of Ile-de-France that brings together most of the health facilities, regardless of their status (public, private or voluntary) (118 facilities), to which were added the primary health care facilities for vulnerable populations (9 facilities). The sampling frame consisting of 127 facilities was stratified by type of facilities and by the number of African migrants in the facility's municipality: primary health care facilities for vulnerable populations constituted a stratum (stratum 1); the FNCS facilities were divided into four strata: the facilities located in municipalities with less than 500 migrants (stratum 2), those in municipalities with between 500 and 800 migrants (stratum 3), those in municipalities between 800 and 1,600 migrants (stratum 4) and finally those in municipalities with more than 1,600 migrants (stratum 5). To obtain 30 participating facilities, 118 were drawn from the sampling frame of 127 facilities thus formed (i.e. facility participation rate of 25% centre). The sampling rate was 0.666 for stratum 1, 0.138 for stratum 2, 0.235 for stratum 3 0.2 stratum 4 and 0.244 for stratum 5. Based on the estimation of the required sample size (set to 1000 people), the sampling rate in each stratum and in each centre was determined according to the weight of the number migrants in each stratum.

Inclusion criteria

- *For the 3 groups*:
  - Be born in a Sub-Saharan African country and with Sub-Saharan African citizenship at birth,
  - Be aged 18 years or older,
  - Followed in the service for one's own health.

- *For the HIV group*:
  - To have been diagnosed for HIV more than 3 months ago, regardless of co-infections

- *For the hepatitis B group:*
  - To have been diagnosed AgHBs+ more than 3 months ago and not be HIV co-infected Patients co-infected with HIV-HBV were thus recruited in the HIV group. Indeed, in cases of HIV and HBV co-infection, HIV medical care is predominant. It is adapted to take into account HBV co-infection (some antiretrovirals used for HIV also have an effect on HBV), but the patient is followed-up within an HIV service. In terms of social impact of the disease, HIV is also predominant. However HBV co-infection was documented and taken into account in the analysis).

- *For the primary care group*:
  - Not be known by the consulting physician as infected by HIV or HBV.

2. Recruitment procedure for patients in each group:

Within each health care facility selected for the study, all health care professionals providing outpatient care to patients born in sub-Saharan Africa were asked to participate in the study. They identified eligible patients during consultations based on medical records, and offered study participation to all eligible patients, except when the patient was deemed unable to be interviewed (for

mental health or physical health reasons or because were unable to be interviewed in one of the languages spoken by the interviewer). When the patient agreed to participate in the study, health care professionals gave her/him an information leaflet on the study and obtained written consent. They also gave the participant a card bearing an identification number to ensure data collection anonymity and confidentiality, and referred her/him to the *Parcours* interviewer at the end of the consultation. The *Parcours* interviewer conducted the interview right after the consultation, in face-to-face mode, in a dedicated room, with closed door to ensure privacy.
The health care professional could also suggest an appointment in the following days, always on site, to patients who agreed to participate in the study but were not immediately available.
The survey participants received a 15€ voucher compensation for the interview time.

Consideration of non-Francophone participants:

To take into account difficulties in participating in the survey due to poor or no knowledge of the French language, the patient questionnaire was available in French or English, and, by appointment, an interpreter could be made available to conduct the interview in an African language spoken by the respondent.

3. Type of data collected

Several data collection tools were used:

Information register

In each service investigated, each health care professional participating in the study held an information register. Each page of the register had two parts, one with a detachable section specifying the correspondence between the identity of study patients and their ID number, and an anonymous one describing the main characteristics of eligible patients. During each consultation day, the health care professional listed in the register all eligible patients, their gender, age group, if the patient had worked at least one day in the previous month, a health indicator (last CD4 count for HIV + patients, last alanine transaminase (ALT) level for patients with hepatitis B, the fact of consulting in this facility for the first time for patients in the reference group), and participation in the survey (participant, not offered participation, refusal).

Life-event history questionnaire

Participants were interviewed using a standardized life-event history questionnaire administered by an interviewer face-to-face (average duration 55 minutes). The life-event questionnaire consisted of two parts:

- A list of questions associated with a **life history calendar** (or biographical grid), which allowed identifying, recording and dating (per year) events in the lives of the respondents, from birth to the date of the survey, and in the various themes explored in the study.
- A **book of thematic modules**, aimed at describing in-depth the events previously identified in the biographical grid (or some of them).

The life history calendar has a very graphic and visual form, which makes it easier to remember, record and date the events in the lives of the respondents. In particular, it encourages and easily adapts to the process of remembering that works by association of ideas and connections between life-event areas ("I went to Mauritania when I got married, I was 19 years old"), and as such optimizes data collection, in terms of completeness and reliability of the data collected. These tools were named the Ageven form (Age-event) and the Life History Calendar (LHC).

The themes explored in the life-event questionnaire were:

- ❖ **Gender, age**

- ❖ **Residential biography and housing history**
- ❖ **Place of birth and places of residence.**
  Changes in residency are identified per year, and periods of residential instability are documented (changes in residence within less than a year). From arrival in France, housing conditions are described in detail, year by year.
- ❖ **Work history - Education and Resources**
  The level of education, type of training and the sequence of activities or periods of inactivity are documented. For each sequence, the type of resources available to the respondent are documented.
- ❖ **Marital and family history**
  Relationships that lasted at least one year are listed. For each relationship we documented the dates of beginning and end of the relationship, if people married or not, if they cohabitated, potential deaths, and the periods of geographic separation. Polygamy situations and the main demographic characteristics of the partner are collected (country of birth, age, educational level), in order to characterize the type of marital relationship.
  The respondent is asked to indicate the moments of her/his life when she/he may have had short-term relationships (less than one year) or casual partners, transactional relationships, and paid sex.
  Finally, we not only list the children born during the relationship, but more broadly all pregnancies in these relationships, and whether they were wanted or not (which is an indicator of the prevention of sexual risks), and their outcome.
- ❖ **History of testing**
  It summarizes all HIV and HBV tests that the respondent may have taken, when, why and what was the result. In case of a positive diagnosis for one and/or the other of these infections, questions are asked on the care schedule after diagnosis, and on disclosure with the entourage, particularly with successive spouses.
- ❖ **General health and history of disease**
  Apart from HIV and HBV, major health problems that may have ever occurred (including at the time of the survey) are described, with specific questions about tuberculosis. Alcohol and tobacco consumption is documented.
- ❖ **Arrival in France and stay in France**
  The time of first arrival in France (that the respondent considers as her/his first settling in France) is more specifically described: reasons for coming to France and arrival conditions. The administrative history from arrival in France is outlined, as well as the history of nationalities if there have been changes. Then medical insurance is documented as well as any problems encountered the health care system in France (financial, administrative, discrimination...).
- ❖ **Sexual and reproductive health**
  A series of more specific questions about sexual and reproductive health are asked: the conditions of sexual debut (age, stress, risk protection) are detailed, as well as the number of sexual partners in lifetime and use of contraception and condoms before and after the arrival in France. Infertility, and potential medical treatments, are identified. Events of potential sexual coercion (forced sex, transactional sex) are characterized. The prevention of sexual risks within the last 12 months is explored in more detail.
- ❖ **Social networks, support networks**
  The list of people cohabiting with the respondent (household) is established. For each household member, participation to household income is stated. The level of well-being in France, the existence or absence of a support network regarding the difficulties encountered in France and in the countries of origin, are explored. For people living with HIV and those with chronic hepatitis B, specific questions are asked about the impact of these infections on the relationship between the person and the entourage.

Medical questionnaire

The health care professional completed the medical questionnaire, from the patient's medical record. For the HIV and hepatitis B groups, this questionnaire documented the chronological landmarks and key parameters of the disease and its treatment. For the reference group, it informed the reason for consultation, potential pathology(ies) at the time of the survey, and current treatments.

Anonymity of data:

The ID number on the anonymity card given by the health care professional to the patient was the only participant identifier for the different survey questionnaires (thematic book and life-event calendar, medical questionnaire). The interviewer did not know the identity of the patient. No personally identifiable data was collected directly or indirectly for eligible individuals not offered participation or individuals refusing study participation. The general characteristics collected on the register among refusals were collected in the form of classes, and not precisely, thus not allowing for later identification.

4. Ethical approvals

The survey was approved by the Advisory Committee for Data Processing in Health Research (CCTIRS) (approval on 13 April 2011) and by the National Commission on Informatics and Liberties (CNIL) (CD-2011-484 approval on 7 December 2011).

5. Data collection results

It was originally planned to recruit, over a period of 9 months, 1000 people in each group HIV, hepatitis B and general medicine, sample size needed to show a 5 to 10% difference between two groups of equal size (statistically significant risk threshold of 5% with a power of 80%) for specific indicators of sexual risk behaviours and social situation. The number of eligible immigrants in the selected health care facilities turned out to be lower than expected, leading to an extended recruitment period. The observational study was conducted for 16 months instead of 9 initially planned. It was discontinued after recruiting 2,500 people, a sample size deemed sufficient to reach satisfactory statistical power, a further extension not being feasible logistically and financially.

Between 30/01/2012 and 31/12/2012, 1,829 individuals infected with HIV and meeting the eligibility criteria presented at the participating services, among which 141 were not offered participation by their physician (111 for health reasons and 30 for language problems). Of the 1,688 individuals offered participation, 762 refused or abandoned during the questionnaire. In total, 926 individuals were included (participation rate: 54.9%). The participation rate was higher among unemployed men compared to those working (60.1% against 49.0%, p=0.05, table 2) but did not differ by sex, age, or CD4 level (tables 1, 2,3).

Between 15/02/2012 and 31/05/2013, 1,169 individuals infected with hepatitis B and meeting the eligibility criteria presented at the participating services, among which 17 were not offered participation by their physician and 8 abandoned during the questionnaire due to language problems. In total, among the 1,135 individuals offered participation, 779 were included (participation rate: 68.5%). The participation rate was higher among men than among women (69.2% against 61.7%, p=0.01, table 4) and among the unemployed compared to those employed (73.0% against 64, 8%, p=0.01, table 4) but did not differ by age or level of transaminases (tables 4,5,6).

Between 15/02/2012 and 31/05/2013, 1,184 meeting the eligibility criteria presented at the participating primary health care facilities. Among these, 124 were not offered participation by their physician (91 for health reasons and 33 for language problems). Among the 1,060 individuals offered participation, 297 refused or abandoned during the questionnaire. In total, 763 individuals were

included (participation rate: 71.9%). Participation did not differ by sex, age and employment status (tables 7,8, 9).

Among all services and groups, the number of refusals due to language problems was 71 (30 HIV, 8 HBV and 33 reference), to which can be added 10 interviews interrupted due to poor understanding of French (8 HBV and 2 reference), i.e. a total of 81 persons among 4,182 (1.94%) who didn't participate in the study for language problems.

6. Weighting

In order to take into account the sample design and non-participation, data was weighted according to each individual's probability of inclusion in the survey (i.e. considering the probability of inclusion in the sample for each health care facility, the number of half-days of weekly consultations in each facility included and the individual study participation per half-day of included consultations).

****

The PARCOURS Study Group included A Desgrées du Loû, F Lert, R Dray Spira, N Bajos, N Lydié (scientific coordinators), J Pannetier, A Ravalihasy, A Gosselin, E Rodary, D Pourette, J Situ, P Revault, P Sogni, J Gelly, Y Le Strat, N Razafindratsima.

Participating centres and investigators :

HIV group :
Hôpital intercommunal de Créteil, service de Médecine Interne (I Delacroix, B El Harrar V Garrait, L Richier) ; CHU le Kremlin Bicêtre, service de Médecine Interne (K Bourdic, M Duracinski, C Goujard, Y Quertainmont, D Peretti) ; Hôpital Delafontaine, service de Maladies Infectieuses (MA Khuong, J Krause, M Poupard, B Taverne, G Tran Van) ; Hôpital Tenon, service de Maladies Infectieuses et Tropicales (A. Adda Lievin, P Bonnard, H Cordel, MG Lebrette, FX Lescure, P Thibault, N Velazquez, JP Vincensini); CHU Lariboisière, service de Médecine Interne A (JF Bergmann, E Badsi, J Cervoni, M Diemer, A Durel, P Sellier, M Bendenoun, JD Magnier) ; CHU Saint-Antoine, service de Maladies Infectieuses et Tropicale (E Bui, J Tredup, P Campa, N Desplanque, M Sebire) ; Hôpital Saint-Louis, services de Maladies Infectieuses et Tropicale et d'Immunologie Clinique (D Ponscarme, N Colin de Verdière, V de Lastours, J Gouguel, C Gatey, C Lascoux-Combe, V Garrait, F Taieb, N De Castro, L Gérard) ; Hôpital Bichat Claude Bernard, service de Maladies Infectieuses, VIH, et Tropicales (G Pahlavan, B Phung, F L'Heriteau, A Dia, A Lepretre, G Morau, C Rioux, V Joly, Y Yazdanpanah, S Legac) ; Hôpital René Dubos, service de Médecine Interne Dermatologie (L Blum, M Deschaud) ; Hôpital Européen George Pompidou, service d'Immunologie Clinique (M.Buisson, P.Kousignian, C.Minozzi, M.Karmochkine, D.Batisse, J. Pavie, M.Eliaczewicz, L.Weiss, G.Gonzalez, P.Castiel, I.Pierre, M.Beumont, D.Jayle, J.Derouinau, M.Manea, E.Bourzam) ; CHU Avicenne, service de Maladies Infectieuses et Tropicales (S Abgral, S Mattioni, R Barruet, N Sayre, O Bouchaud, M Tatay, M Gousseff, F Mechai, T Kandel, P Honoré, M Coupard) ; CHU Cochin Tannier, service d'unité Fonctionnelle de Pathologie Infectieuse (F.Bani-Sadr, L.Belarbi, G.Spiridon, T.Tahi, H.Mehawej, D.Salmon, B.Silbermann, A.Calboreanu, P.Loulergue, H.Bouchez, V.Le Baut, M.P.Pietri) ; Centre hospitalier de Gonesse, service de Médecine Interne (D.Trois Vallets, V.Favret) ; Hôtel Dieu, Centre de Diagnostic et de Thérapeutique (JP Viard, A Sobel, J Gilquin, A Cros, A Maignan) ; CHI Poissy/Saint-Germain-en-Laye, service de Maladies Infectieuses et Tropicales (Y Welker, H Masson, B Montoya, JL Ecobichon) ; CH de Lagny Marne la Vallée, service de maladies infectieuses (E Froguel, P Simon, MS Nguessan, S Tassi) ; Nouveau Centre Hospitalier Sud Francilien, service d'Hématologie Clinique (A Devidas, L Gerard, H Touahri) ; CHI Robert Ballanger, service de Médecine Interne et Maladies Infectieuses (JL Delassus, MA Bouldouyre, V Favret) ; Hôpital Intercommunal André Grégoire, service de Médecine Interne (C Winter) ; CHU La Pitié-Salpetrière, services de Médecine Interne 1 et de Maladies Infectieuses et Tropicales (S Pelletier, MC Samba, S Herson, A Simon, M Bonmarchand, N Amirat, N Smail, S

Seang, H Stitou, L Schneider, R Tubiana, L Epelboin, MA Valantin, C Katlama, S Ben Abdallah, P Bourse, Y Dudoit, C Blanc) ; Centre Médical Institut Pasteur, consultations spécialisées (C Charlier-Woerther, O Lortholary, PH Consigny, A Gergely, M Shoai-Tehrani, G Cessot, C Duvivier, F Touam, K Benhadj) ; CMC de Bligny, service de Maladies Infectieuses et VIH (H Ait Mohand, E Klement, P Chardon).

Chronic Hepattitis B group :
Hôpital Beaujon, service d'Hépatologie (A Pumpo, T Asselah, N Guilly, N Boyer, F Mouri, C Castelnaud, M Pouteau) ; Hôpital Pitié-Salpêtrière, service d'Hépato-Gastroentérologie et de Médecine Interne (T Poynard, Y Benhamou, G Bonyhay, P Lebray, J Massard, J Moussali, V Ratziu, M Rudler, D Thabut, R Pais, E Luckina, A Simon, MC Samba, S Pelletier, N Smail) ; Hôpital Paul Brousse, Centre Hépato-Biliaire (JC Duclos Vallée, D Samuel, A Coilly) ; CHU Henri Mondor, service d'Hépatologie (F Roudot-Thoraval, C Hezode, E Lopes) ; Hôpital Jean Verdier, service d'Hépato-Gastroentérologie (V Bourcier, N Ganne, P Nahon, J Cohelo, JC Trinchet, G Gnkontchou, V Grando, S Brulé) ; Hôpital Cochin, unité d'Hépatologie (P Sogni, H Fontaine, V Mallet, S Tripon, A Vallet Pichard, S Pol, M Corouge, M Benazra, A Noble, L Bousquet) ; Hôpital Saint-Antoine, service d'Hépato-Gastroentérologie (D Mohand, L Serfaty, N Carbonell, C Corpechot, M Lequoy, H Mathiex-Fortunet, A Pascale, O Rosmorduc, O Chazouillères, S Mezhoud, T Andreani, H Regnault, A Poujol-Robert, F Benjelloun) ; Hôpital Gouin, service d'Hépato-Gastroentérologie (S Levy) ; Hôpital Avicenne, services de Médecine Interne et de Maladies Infectieuses (D Roulot, U Warzocha, C Cambonie, O Bouchaud, F Mechai, M Goussef, N Vignier, T Kandel, P Honore) ; CH de Lagny Marne La Vallée, service de Gastroentérologie (F Harnois, G Macaigne, K Fernandes) ; Hôpital Lariboisière, services de Médecine Interne et d'Hépato-Gastroentérologie (JF Bergmann, P Sellier, M Bendenoun, JD Magnier, V Ozenne) ; Hôpital Tenon, services d'Hépato-Gastroentérologie et de Maladies Infectieuses (D Ancel, I Popa-Gregoriou, F Amiot, A Garioud, JD Grangé, D Chaslin-Ferbus, S Gil-Diez, P Bonnard, MG Lebrette, H Cordel, FX Lescure, JP Vincensini, A Adda Lievin, P Thibault, N Velazquez) ; Centre Hospitalier Intercommunal de Créteil, service d'Hépato-Gastroentérologie (S Barge, I Rosa, L Costes, L Richier) ; Réseau ARES 92 (A Seif, C Chandemerle, M Lalande, T Mazars) ; Réseau Paris Nord (JP Aubert, O Taulera) ; Centre de santé du Comede (I Alix, F Fleury, O Lefebvre, P Revault, M Petruzzi, C Menard).

Primary care group :
Centre municipal de santé Gentilly (JJ Bourcart) ; PASS Hôpital Saint-Louis (C Georges, M Andlauer, Y Girardeau, F Tapié de Celeyran, P Bordon, N Dardel, D Levy, O Taulera, ML Pintir Rageau, S Dehaudt) ; PASS Hôpital Lariboisière (C Aparicio, U Loeung, C Toussaint) ; PASS Hôpital Saint-Antoine (V Vasseur, A Andel, O Cha, P Xu, V Sivalingam, I Hiesse, S Ouaret, L Zahzam, E Bui) ; PASS hôpital La Pitié-Salpêtrière (E Baut, MA Temple, A Gillet, C Leata, J Legoff) ; Centre médical et dentaire Henri Barbusse-Gagny (F Jardin, E Jean Baptiste) ; Centre d'Accueil de Soins et d'Orientation (E Corp, J Nau, B Chatelin, P Weeger, N Hervier, L Lacroze) ; Centre municipal de santé Colombes (L Ballonard, A.Seif) ; Centre médico-social COMEDE (I Alix, F Fleury, O Lefebvre, P Revault, M Petruzzi, C Menard) ; Centre médico-social Fresnes (Y Chahan, J Prost) ; Centre médico-social Livry-Gargan (D Spindler, L Rodin Zita, Nguyen, K Dambielle) ; Centre médico-social Ambroise Croizat Franconville (P Bourlier, P Zaidi) ; Centre médico-social Mai-Politzer Arcueil (F Block) ; Centre de santé Françoise Dolto Tremblay-en-France (N Bessah) ; Centre médico-social Fontenay aux roses (G Barbier, G Pilorget, N Touazi) ; Centre Polyvalent de Santé Aimé Césaire Bobigny (F Rouges, C Chalal) ; Centre médical Pierre Rouques Villejuif (L Cavanna, A Sabah, M C Charansonnet, G Ibanez) ; Centre de santé C.P.A.M. Choisy-le-Roi (M Lesourd) ; Centre municipal de santé Issy-les-Moulineaux (M Orbach-Roulière, JP Vilanova) ; Centre médical Elio Habib -Paris (M Smadja, E Rozenkier) ; Centre de santé Pierre Rouquès Le Blanc-Mesnil (C Vincent, V Bontemps, A Ngaleu, F Bensidhoum) ; Centre de santé Centre médico-social JA Dolidier Pierrefitte (M Gharmaoui, B Hubert-Andreani, V Vinçon, K Royer, S Vernerey-Martin, C Meunier, S Djouhri, V Ngadjeu) ; Centre médico-social Maurice Ténine Champigny-sur-Marne (F Villebrun, D Soletchnik, M Deroche, S Henry, M Geminel, C Tison, M Dupuy, E Balgone) ; Centre municipal de santé Nanterre (M Esteve, C Terra, Charles, L Rossignol, C Bismuth, G Gillemot, M Vanbana) ; Centre de santé Henri Barbusse Saint-Ouen (M Djouab, G Lyon, F Ogier, J.Gelly) ; Centres de santé Savattero Montreuil (P Vidigal,

A Gibier, A Simonnet-Richard, C Alvarez-Malgras) ; Dispensaire C.R.F. Boulogne Billancourt (C Dufresne, B Huault, I Cornuche) ; Centre municipal de Santé Louis-Fernet Sevran (H Touitou, S D'Epiro, F Michard, N Guesnier, F Siecat) ; Centre médico-social Haxo Paris (N Pinard, H Gaborieau) ; Centre médico-social Pierre Rouques Vitry-sur-Seine (A Beaupin, G Albouy, C Besacier, A Soares, J Le Breton).

ANNEXES

Table 1: Factors associated to study participation in the HIV group

		Univariate	Multivariate	
	N	% [IC95%]	OR [95% IC]	p-value
Sex				
Women	1158	51,1 [42,6-59,6]	- 1 -	*Ref.*
Men	668	51,9 [39,1-57,3]	1,01 [0,72 ; 1,43]	0,95
Age				
< 30	131	48,1 [34,8-61,6]	0,87 [0,53 ; 1,41]	0,55
30 - 39	608	52,4 [43,2-61,5]	0,95 [0,76 ; 1,20]	0,66
40 - 49	647	53,7 [44,9-62,2]	- 1 -	*Ref.*
50 - 59	382	52,5 [42,9-61,8]	0,90 [0,69 ; 1,18]	0,44
CD4 cells count				
<200	150	61,7 [46,6-74,8]	1,39 [0,86 ; 2,27]	0,17
[200 ; 349]	306	55,9 [48,1-63,5]	1,21 [0,97 ; 1,50]	0,08
[350 ; 499]	448	52,9 [44,5-61,1]	1,07 [0,89 ; 1,28]	0,44
≥500	850	49,8 [40,7-58,9]	- 1 -	*Ref.*
Employment situation				
With employment	1135	48,7 [38,2-59,2]	- 1 -	*Ref*
Without employment	533	59,4 [52,8-65,7]	1,57 [0,95 ; 2,60]	0,08

weighted percentages
*p<0.05 **p<0.01 *p-value: design-based* x^2 *test*
OR : Odds Ratio adjusted for sex, age, CD4 cells count and employment situation
3 missing values on the variable sex in the HIV group

Table 2: Factors associated to study participation among men in the HIV group

		Univariate	Multivariate	
	N	% [IC95%]	OR [95% IC]	p-value
Age				
< 30	32	44,6 [23,2-68,3]	0,90 [0,39 ; 2,07]	0,79
30 - 39	157	51,2 [40,6-61,6]	1,00 [0,71 ; 1,40]	0,99
40 - 49	236	52,7 [43,7-61,7]	- 1 -	*Ref.*
50 - 59	223	55,7 [43,5-67,2]	1,08 [0,74 ; 1,57]	0,66
CD4 cells count				
<200	259	61,4 [42,1-77,6]	1,34 [0,69 ; 2,61]	0,36
[200 ; 349]	172	56,0 [49,2-62,6]	1,14 [0,67 ; 1,94]	0,61
[350 ; 499]	133	53,8 [44,8-62,6]	1,10 [0,71 ; 1,68]	0,66
≥500	74	49,4 [35,7-63,1]	- 1 -	*Ref.*
Employment situation				
With employment	463	49,0 [38,6-59,4]	- 1 -	*Ref.*
Without employment	159	60,1 [48,8-70,4]	1,67 [1,00 ; 2,78]	0,049*

weighted percentages
*p<0.05 **p<0.01 *p-value: design-based* x^2 *test*
OR : Odds Ratio adjusted for age, CD4 cells count and employment situation

Table 3: Factors associated to study participation among men in the HIV group

		Univariate	Multivariate	
	N	% [IC95%]	OR [95% IC]	p-value
Age				
< 30	99	49,2 [34,8-63,8]	0,85 [0,47 ; 1,53]	0,56
30 - 39	451	52,8 [43,1-62,4]	0,92 [0,70 ; 1,20]	0,52
40 - 49	411	54,2 [43,7-64,2]	- 1 -	*Ref.*
50 - 59	159	47,9 [39,1-56,9]	0,74 [0,53 ; 1,04]	0,08
CD4 cells count				
<200	76	61,9 [45,5-76,0]	1,41 [0,81 ; 2,47]	0,21
[200 ; 349]	173	55,9 [44,5-66,7]	1,25 [0,88 ; 1,79]	0,20
[350 ; 499]	276	52,4 [42,3-62,2]	1,04 [0,77 ; 1,41]	0,76
≥500	591	50,0 [41,4-58,6]	- 1 -	*Ref.*
Employment situation				
With employment	672	48,5 [36,8-60,4]	- 1 -	*Ref.*
Without employment	374	59,1 [51,6-66,1]	1,53 [0,84 ; 2,79]	0,16

weighted percentages

*p<0.05 **p<0.01 *p-value: design-based* x^2 *test*
OR : Odds Ratio adjusted for age, CD4 cells count and employment situation

Table 4: Factors associated to study participation in the CHB group

		Univariate	Multivariate	
	N	% [IC95%]	OR [95% IC]	p-value
Sex				
Women	356	61,7 [51,1-71,2]	- 1 -	*Ref.*
Men	808	69,2 [60,0-77,2]	1,55 [1,11 ; 2,17]	0,01*
Age				
< 30	194	66,6 [54,6-76,7]	0,76 [0,54 ; 1,07]	0,10
30 - 39	451	67,6 [55,3-77,9]	0,90 [0,65 ; 1,26]	0,53
40 - 49	315	71,5 [62,4-79,2]	- 1 -	*Ref.*
50 - 59	154	70,4 [54,6-82,5]	0,90 [0,56 ; 1,43]	0,63
Hepatic transaminases				
1 – 3 N	995	72,1 [59,1-82,3]	- 1 -	*Ref.*
+ 3 N	25	59,2 [40,0-77,5]	0,68 [0,28 ; 1,68]	0,38
Employment situation				
With employment	669	64,8 [51,5-76,1]	- 1 -	*Ref.*
Without employment	318	73,0 [62,9-81,2]	1,57 [1,12 ; 2,21]	0,01*

weighted percentages
*p<0.05 **p<0.01 *p-value: design-based* x^2 *test*
OR : Odds Ratio adjusted for sex, age, level of transaminases and employment situation
4 missing values on the variable sex in the CHB group

Table 5: Factors associated to study participation among men in the CHB group

		Univariate	Multivariate	
	N	% [IC95%]	OR [95% IC]	p-value
Age				
< 30	121	70,5 [56,1-81,8]	0,80 [0,42 ; 1,54]	0,49
30 - 39	313	69,3 [57,9-78,7]	0,86 [0,60 ; 1,23]	0,40
40 - 49	226	72,6 [62,7-80,6]	- 1 -	*Ref.*
50 - 59	117	74,0 [56,7-86,1]	1,00 [0,47 ; 2,13]	0,99
Hepatic transaminases				
1 – 3 N	705	74,9 [62,0-84,5]	- 1 -	*Ref.*
+ 3 N	14	53,5 [32,2-73,6]	0,50 [0,19 ; 1,31]	0,15
Employment situation				
With employment	496	65,7 [61,3-77,6]	- 1 -	*Ref.*
Without employment	184	81,0 [71,7-87,8]	2,25 [1,39 ; 3,64]	0,003*

weighted percentages
*p<0.05 **p<0.01 *p-value: design-based* x^2 *test*
OR : Odds Ratio adjusted for age, level of transaminases and employment situation

Table 61: Factors associated to study participation among women in the CHB group

		Univariate	Multivariate	
	N	% [IC95%]	OR [95% IC]	p-value
Age				
< 30	73	60,0 [42,5-75,2]	0,66 [0,40 ; 1,09]	0,10
30 - 39	138	64,1 [48,6-77,1]	0,91 [0,56 ; 1,48]	0,69
40 - 49	90	69,0 [59,2-77,3]	- 1 -	*Ref.*
50 - 59	35	63,5 [42,6-80,3]	0,56 [0,27 ; 1,17]	0,11
Hepatic transaminases				
1 – 3 N	290	65,7 [51,7-77,4]	- 1 -	*Ref.*
+ 3 N	11	67,0 [31,9-89,8]	0,92 [0,25 ; 3,45]	0,90
Employment situation				
With employment	173	62,2 [50,9-72,4]	- 1 -	*Ref.*
Without employment	134	62,6 [48,3-75,1]	0,95 [0,67 ; 1,35]	0,76

weighted percentages
*p<0.05 **p<0.01 *p-value: design-based* x^2 *test*
OR : Odds Ratio adjusted for age, and employment situation

Table 72: Factors associated to study participation in the MG group

		Univariate	Multivariate	
	N	% [IC95%]	OR [95% IC]	p-value
Sex				
Women	637	69,1 [58,9-77,7]	- 1 -	*Ref.*
Men	545	71,3 [62,7-78,5]	1,14 [0,84 ; 1,55]	0,39
Age				
< 30	279	69,1 [54,7-80,6]	0,95 [0,58 ; 1,55]	0,82
30 - 39	356	69,2 [56,3-79,7]	0,90 [0,60 ; 1,36]	0,61
40 - 49	310	71,2 [62,7-78,5]	- 1 -	*Ref.*
50 - 59	207	79,5 [70,1-86,5]	1,45 [0,97 ; 2,16]	0,07
Employment situation				
With employment	601	73,9 [66,5-80,1]	- 1 -	*Ref.*
Without employment	543	65,7 [49,7-78,7]	0,73 [0,43 ; 1,23]	0,23

weighted percentages
*p<0.05 **p<0.01 *p-value: design-based* x^2 *test*
OR : Odds Ratio adjusted for sex, age, level of transaminases and employment situation
3 missing values on the variable sex in the MG group

Table 8: Factors associated to study participation among men in the MG group

		Univariate	Multivariate	
	N	% [IC95%]	OR [95% IC]	p-value
Age				
< 30	123	72,4 [56,8-83,9]	0,92 [0,54 ; 1,57]	0,76
30 - 39	162	69,3 [55,6-80,2]	0,77 [0,46 ; 1,28]	0,30
40 - 49	144	75,5 [67,3-82,1]	- 1 -	*Ref.*
50 - 59	103	76,4 [66,6-84,0]	1,06 [0,64 ; 1,74]	0,82
Employment situation				
With employment	302	74,2 [65,0-81,6]	- 1 -	*Référence*
Without employment	224	69,7 [54,1-81,8]	0,84 [0,43 ; 1,64]	0,59

weighted percentages
*p<0.05 **p<0.01 *p-value: design-based* x^2 *test*
OR : Odds Ratio adjusted for age and employment situation

Table 9: Factors associated to study participation among women in the MG group

		Univariate	Multivariate	
	N	% [IC95%]	OR [95% IC]	p-value
Age				
< 30	156	66,9 [49,5-80,7]	0,97 [0,44 ; 2,16]	0,94
30 - 39	194	69,2 [55,1-80,4]	1,00 [0,60 ; 1,69]	0,97
40 - 49	165	68,4 [58,2-77,1]	- 1 -	*Ref.*
50 - 59	104	82,0 [69,6-90,0]	1,88 [0,96 ; 3,67]	0,06
Employment situation				
With employment	299	73,6 [65,3-80,5]	- 1 -	*Ref.*
Without employment	318	63,7 [46,2-78,2]	0,67 [0,36 ; 1,22]	0,18

weighted percentages
*p<0.05 **p<0.01 *p-value: design-based* x^2 *test*
OR : Odds Ratio adjusted for age and employment situation
