# Supplementary material for: "Times Are Changing": The Impact of HIV Diagnosis on Sub-Saharan Migrants’ Lives in France
Source: PLoS One. 2017 Jan 27;12(1):e0170226. doi: 10.1371/journal.pone.0170226 (PMC5271323; doi:10.1371/journal.pone.0170226)
Supplement: S2 File — (DOCX) [file pone.0170226.s002.docx]

**S2 File. Construction of curves of Figure 1 and Figure 2**

These curves show the proportion of persons in activity, in union and in perceived well-being in relation to two key moments: migration and diagnosis, for persons who were diagnosed after migration.

For each person, we divide the lifetime into three periods: i) before migration, ii) between migration and diagnosis, and iii) after diagnosis. Each period is divided in 20 quantiles, so it allows us to calculate for instance the rate of activity at 5%, 10%, 15%... of each period for each individual.

We then produce tables of the activity, union and well-being rates for each subgroup, for each of the three periods. The graphical construction hereby obtained allows to characterise the distribution of the three lifetime indicators in relation with these two relative moments of migration and diagnosis.

The curves are smoothed thanks to weighted moving average method.
