# Supplementary material for: "Times Are Changing": The Impact of HIV Diagnosis on Sub-Saharan Migrants’ Lives in France
Source: PLoS One. 2017 Jan 27;12(1):e0170226. doi: 10.1371/journal.pone.0170226 (PMC5271323; doi:10.1371/journal.pone.0170226)
Supplement: S3 File — (DOCX) [file pone.0170226.s003.docx]

S3 File. STROBE Statement—Checklist of items that should be included in reports of ***cross-sectional studies***

***Applied to the PARCOURS Study***

|  | **Item N°** | **Recommendation** | **Pages** |
| --- | --- | --- | --- |
| **Title and abstract** | 1 | 1. Indicate the study’s design with a commonly used term in the title or the abstract   **(Abstract) : “We use the ANRS PARCOURS survey, a retrospective life-event survey led in 2012-2013… »** | Manuscript p.2 |
|  |  | 1. Provide in the abstract an informative and balanced summary of what was done and what was found   ***See abstract*** | Manuscript p.2 |
| **Introduction** |  |  |  |
| Background/rationale | 2 | Explain the scientific background and rationale for the investigation being reported  **(Introduction) Migrants living in Europe account for 35% of HIV incident diagnoses of HIV/AIDS (1) and persons coming from Sub-Saharan Africa represented 31% of new HIV diagnoses in France in 2013 (2). HIV/AIDS may entail a biographical disruption in people’s lives (3) and impact many spheres, such as patients’ professional lives, intimate ties, social networks, sexuality, and more generally life expectations and well-being. This was especially the case at the beginning of the AIDS era, when an HIV-positive diagnosis was tantamount to a death sentence. Previous work has shown that HIV-infection negatively affected persons’ chances of accessing employment, particularly among women (3–5). It was also shown that persons living with HIV (PLHIV) could experience negative reactions from their partners and spouse when they learned about their infection (6) as well as within the family circle (7). Migrants arriving in France have already experienced a disruption of their lives because they have left their home countries. Indeed, the migration pathway often has its pitfalls, provokes long-lasting separations of families (8) and causes occupational downgrading (9,10). What, then, is the impact of HIV diagnosis on lives that are already disrupted by migration?**  **(…)Thus, the respective impacts of these two diseases can be questioned: even if HIV/AIDS benefits from an exceptional mobilization in comparison to chronic Hepatitis B, its normalization as a ‘chronic disease like any other’ may be challenged by health complications, decreased self-esteem, stigmatization and discrimination as well as social hardships that PLHIV may experience.** | Manuscript p.3-4 |
| Objectives | 3 | State specific objectives, including any prespecified hypotheses :  **(Introduction) :** **The objective of this paper is to assess the impact of HIV diagnosis on the lives of Sub-Saharan migrants living in France using data from a large life-event survey of people from sub-Saharan Africa living in France: the ANRS PARCOURS survey. We analyze the respective impacts of HIV diagnosis and migration, and by studying diagnoses before and after the arrival of ARTs, we wish to question the ‘normalization’ of life with HIV.**  *Prespecified hypothesis :* **1) Previous work has shown that HIV-infection negatively affected persons’ chances of accessing employment, particularly among women 2) With the progressive transformation of HIV/AIDS into a chronic illness, the social impact of HIV/AIDS may have changed. The debate about whether HIV/AIDS could be a ‘chronic disease like any other’ has been ongoing since the arrival of effective treatments in 1996 ; 3) HIV/AIDS benefits from an exceptional mobilization (Smith & Whiteside 2010) in comparison to chronic Hepatitis B.** | Manuscript p.3-4 |
| **Methods** |  |  |  |
| Study design | 4 | **Present key elements of study design early in the paper :**  **(Introduction) The objective of this paper is to assess the impact of HIV diagnosis on the lives of Sub-Saharan migrants living in France using data from a large life-event survey of people from sub-Saharan Africa living in France: the ANRS PARCOURS survey.**  **(Material and Methods) The ANRS PARCOURS study was conducted to analyze how health trajectories and social and migratory paths are interlaced for migrants from Sub-Saharan Africa living in France. This retrospective quantitative life-event survey was conducted from February 2012 to May 2013 (..)** | Manuscript p.3  p.4(end) |
| Setting | 5 | Describe the setting, locations, and relevant dates, including periods of recruitment, exposure, follow-up, and data collection  **(Material and Methods) This retrospective quantitative life-event survey was conducted from February 2012 to May 2013 in healthcare facilities in the greater Paris metropolitan area (Ile-de-France) among two groups of migrants born in Sub-Saharan Africa: one receiving HIV care, one with chronic hepatitis B (and not HIV infected).** | Manuscript p.5 |
| Participants | 6 | *(*a) Give the eligibility criteria, and the sources and methods of selection of participants  **(Material and Methods) Patients were eligible if they were born in Sub-Saharan Africa, aged 18 to 59, and diagnosed at least 3 months earlier. HIV and Hepatitis B groups were randomly sampled and are representative of migrants followed for these pathologies in the Paris metropolitan area. All information was anonymously collected. (…).**  **Physicians asked all eligible patients, except those with major cognitive or health impairments, to participate and collected their written consent.** | Manuscript p.5 |
| Variables | 7 | Clearly define all outcomes, exposures, predictors, potential confounders, and effect modifiers. Give diagnostic criteria, if applicable.  **(Material and Methods) *Outcomes and variables of interest* :**  **To consider the impact of diagnosis on both living conditions and well-being, we looked at three outcomes for each year between 18 years of age and the date of data collection: being in activity, being in union and perceived well-being.**  **Being in activity (yes/no for a given year) means having a job (regardless of the job being in the formal sector or not) or being a student during most of the year considered. Being in union (yes/no for a given year) is defined as being in a relationship that lasted at least a year, regardless of marital status or whether ego lives with his or her partner. Eventually, the persons were asked to indicate which periods in their life they considered as ‘good years’, ‘difficult years’ or ‘neither good nor bad years’. Well-being (yes/no for a given year) is then defined as a year mentioned as a ‘good year’ or a ‘neither good nor bad year’.**  **To look at the impact of diagnosis (either HIV or chronic hepatitis B), we distinguished in each patient’s life among the period before diagnosis, the period just after diagnosis (year of the diagnosis and year after) and the period after. To look at the impact of migration, we distinguished in each patient’s life among the period before migration, the period just after migration (year of migration and year after) and the period after.**  **To account for dramatic changes in the prognosis of HIV infection thanks to advances in ART, we considered three different periods: before 1996 (the arrival of effective ART (Antiretroviral Therapy)), between 1996 and 2004 (when access to treatment existed in France but not in Sub-Saharan Africa) and after 2004, when the scale-up of treatments began in Sub-Saharan Africa, although important issues remain regarding access (UNAIDS 2014).**  ***Control variables***  **All the models are adjusted on socio-demographic characteristics of the interviewees, which can influence their migratory trajectory. These include region of origin (West Africa, Central Eastern and Southern Africa), education level (none or primary, secondary, superior), and reasons for migration (take a chance, join a family member, threatened in country of origin, study, medical reasons). Two time-dependent variables were included : age in four categories (18-24; 25-34; 35-44; 45-59) and to have a child birth in a given year (yes/no).** | Manuscript p.6-7 |
| Data sources/measurement | 8* | For each variable of interest, give sources of data and details of methods of assessment (measurement). Describe comparability of assessment methods if there is more than one group  ***The variables of interest were all collected through a life event questionnaire.***  **Participants were interviewed using a standardized life-event history questionnaire administered by an interviewer face-to-face (average duration 55 minutes). The life-event questionnaire consisted of two parts: - A list of questions associated with a life history calendar (or biographical grid), which allowed identifying, recording and dating (per year) events in the lives of the respondents, from birth to the date of the survey, and in the various themes explored in the study. - A book of thematic modules, aimed at describing in-depth the events previously identified in the biographical grid (or some of them). The life history calendar has a very graphic and visual form, which makes it easier to remember, record and date the events in the lives of the respondents. In particular, it encourages and easily adapts to the process of remembering that works by association of ideas and connections between life-event areas ("I went to Mauritania when I got married, I was 19 years old"), and as such optimizes data collection, in terms of completeness and reliability of the data collected. These tools were named the Ageven form (Age-event) and the Life History Calendar (LHC).** | Protocole p.4 |
| Bias | 9 | **Describe any efforts to address potential sources of bias :**  **(Material and Methods) *Control variables***  **All the models are adjusted on socio-demographic characteristics of the interviewees, which can influence their migratory trajectory. These include region of origin (West Africa, Central Eastern and Southern Africa), education level (none or primary, secondary, superior), and reasons for migration (take a chance, join a family member, threatened in country of origin, study, medical reasons). Two time-dependent variables were included : age in four categories (18-24; 25-34; 35-44; 45-59) and to have a child birth in a given year (yes/no). (…)**  **Data were weighted according to each individual's probability of inclusion in the survey.** | Manuscript p.7-8 |
| Study size | 10 | Explain how the study size was arrived at :  **(Parcours Protocol, supplementary file): It was originally planned to recruit,** ov**er a period of 9 months, 1000 people in each group HIV, hepatitis B and general medicine, sample size needed to show a 5 to 10% difference between two groups of equal size (statistically significant risk threshold of 5% with a power of 80%) for specific indicators of sexual risk behaviours and social situation. The number of eligible immigrants in the selected health care facilities turned out to be lower than expected, leading to an extended recruitment period. The observational study was conducted for 16 months instead of 9 initially planned. It was discontinued after recruiting 2,500 people, a sample size deemed sufficient to reach satisfactory statistical power, a further extension not being feasible logistically and financially.** | Protocol p.6 |
| Quantitative variables | 11 | Explain how quantitative variables were handled in the analyses. If applicable, describe which groupings were chosen and why.    ***Outcome variables : See point 7***  **The associations year by year (since 18 years of age) between transition towards inactivity and the situation regarding migration and diagnosis were analyzed with discrete-time logistic regression models for recurrent events, per sex and study group (HIV group and Hepatitis B group).** | Manuscript p.6  Manuscript p.7-8 |
| Statistical methods | 12 | *(*a) Describe all statistical methods, including those used to control for confounding  **(Material and Methods) We approached the measure of impact in two ways: first we looked at the proportions of persons in activity, union and well-being over time, and then we focused the analysis on the probability to experience an activity loss, conjugal break up and well-being degradation and the factors associated with these outcomes.**  **To compare the respective impacts of HIV diagnosis and migration, we described the distribution of the proportions of persons in activity, in union and with perceived well-being over time, in relation to migration and diagnosis, by group and sex thanks to relative-time graphs (19) (See Supporting Information 2). These curves allow us to see how the proportions vary over time and what the respective impacts of migration and HIV diagnosis are. We then described with similar curves the respective impacts of migration and diagnosis of chronic Hepatitis B.**  **To observe whether the impact of HIV diagnosis has changed over time, we compared with a paired chi2-test the proportions of persons who were in activity, in union and with perceived well-being the year before diagnosis and the year after, by period of HIV diagnosis (before 1996, 1996-2004 and after 2004). We performed the same analysis for chronic Hepatitis B diagnosis.**  **Secondly, we modeled the probability of losing activity (transition from activity or studies towards inactivity), of conjugal break ups and of losing well-being (transition from ‘good years’ or ‘neither good nor bad years’ towards ill-being). The associations year by year (since 18 years of age) between transition towards inactivity and the situation regarding migration and diagnosis were analyzed with discrete-time logistic regression models for recurrent events, per sex and study group (HIV group and Hepatitis B group). Multivariate models were adjusted for time-independent variables (region of origin, education level, reasons for migration) and also for time-dependent variables (age, period, child birth). The same models were used to look at the factors associated with conjugal break-up and transition from well-being towards ill-being.** | Manuscript p.7-8 |
|  |  | *(*b) Describe any methods used to examine subgroups and interactions  ***Interactions were checked. All the Odds ratios went in the same way and interactions did not jeopardize our results.*** |  |
|  |  | *(*c) Explain how missing data were addressed  Missing data were excluded :  **(Results) In total, 1705 persons were included in the HIV and Hepatitis B groups in the PARCOURS survey (926 with HIV and 779 with chronic hepatitis B). A total of 601 had been diagnosed less than five years earlier, 131 were diagnosed or had migrated before 18 years of age, 118 had been diagnosed before migration and for 22 persons there were missing data. Ultimately, 833 persons were included in the study (505 HIV-positive, 328 with chronic Hepatitis B).** | Manuscript  p.8 |
|  |  | (d) If applicable, describe analytical methods taking account of sampling strategy  **(Protocol) In order to take into account the sample design and non-participation, data was weighted according to each individual's probability of inclusion in the survey (i.e. considering the probability of inclusion in the sample for each health care facility, the number of half-days of weekly consultations in each facility included and the individual study participation per half-day of included consultations).**  (methods) **Data were weighted according to each individual’s probability of inclusion in the survey, and the weights applied to all percentages** | Protocol p. 7  Manuscript p.8 |
|  |  | (*e*) Describe any sensitivity analyses  **Not** **applicable** |  |
| **Results** |  |  |  |
| Participants | 13* | (a) Report numbers of individuals at each stage of study—eg numbers potentially eligible, examined for eligibility, confirmed eligible, included in the study, completing follow-up, and analysed  **(Protocol) Between 30/01/2012 and 31/12/2012, 1,829 individuals infected with HIV and meeting the eligibility criteria presented at the participating services, among which 141 were not offered participation by their physician (111 for health reasons and 30 for language problems). Of the 1,688 individuals offered participation, 762 refused or abandoned during the questionnaire. In total, 926 individuals were included (participation rate: 54.9%). The participation rate was higher among unemployed men compared to those working (61.3% against 50.1%, p=0.008) but did not differ by sex, age, or CD4 level.**  **Between 30/01/2012 and 31/12/2012, 1,168 individuals infected with hepatitis B and meeting the eligibility criteria presented at the participating services, among which 17 were not offered participation by their physician and 8 abandoned during the questionnaire due to language problems. In total, among the 1,135 individuals offered participation, 778 were included (participation rate: 68.5%). The participation rate was higher among men than among women (69.2% against 61.7%, p=0.02) and among the unemployed compared to those employed (73.0% against 64, 8%, p=0.03) but did not differ by age or level of transaminases.**  **Between 30/01/2012 and 31/12/2012, 1,185 meeting the eligibility criteria presented at the participating primary health care facilities. Among these, 124 were not offered participation by their physician (91 for health reasons and 33 for language problems). Among the 1,061 individuals offered participation, 297 refused or abandoned during the questionnaire. In total, 763 individuals were included (participation rate: 71.9%). Participation did not differ by sex, age and employment status.**  **Among all services and groups, the number of refusals due to language problems was 71 (30 HIV, 8 HBV and 33 reference), to which can be added 10 interviews interrupted due to poor understanding of French (8 HBV and 2 reference), i.e. a total of 81 persons among 4,182 (1.94%) who didn’t participate in the study for language problems.** | Protocol p.6 |
|  |  | (b) Give reasons for non-participation at each stage  ***See 13a.*** |  |
|  |  | (c) Consider use of a flow diagram  ***As it is a cross-sectional study and not a follow-up, we considered it was not necessary.*** |  |
| Descriptive data | 14* | (a) Give characteristics of study participants (eg demographic, clinical, social) and information on exposures and potential confounders  ***See table 1*** | Manuscript p.9 |
|  |  | (b) Indicate number of participants with missing data for each variable of interest  ***See 12.c*** |  |
| Outcome data | 15* | Report numbers of outcome events or summary measures  ***See table 1*** | Manuscript p.9 |
| Main results | 16 | (a) Give unadjusted estimates and, if applicable, confounder-adjusted estimates and their precision (eg, 95% confidence interval). Make clear which confounders were adjusted for and why they were included  **(Methods) All the models are adjusted on socio-demographic characteristics of the interviewees, which can influence their migratory trajectory. These include region of origin (West Africa, Central Eastern and Southern Africa), education level (none or primary, secondary, superior), and reasons for migration (take a chance, join a family member, threatened in country of origin, study, medical reasons). Two time-dependent variables were included : age in four categories (18-24; 25-34; 35-44; 45-59) and to have a child birth in a given year (yes/no).** | Manuscript p.7 |
|  |  | **(b) Report category boundaries when continuous variables were categorized**  **Two time-dependent variables were included : age in four categories (18-24; 25-34; 35-44; 45-59)…** | Manuscript p.7 |
|  |  | *(*c) If relevant, consider translating estimates of relative risk into absolute risk for a meaningful time period  ***Not applicable*** |  |
| Other analyses | 17 | Report other analyses done—eg analyses of subgroups and interactions, and sensitivity analyses  ***See 12b.*** |  |
| **Discussion** |  |  |  |
| Key results | 18 | Summarise key results with reference to study objectives  **(Discussion) : In this study, we aimed at assessing the impact of HIV diagnosis on activity and union status (objective experience) and also on well-being (subjective experience). The results show that HIV diagnosis does not impact on activity nor on union, at a population level, but it has a strong negative impact on well-being which still exists, even though it tends to diminish after the generalization of effective ARTs. In comparison, Hepatitis B diagnosis did not have any significant impact of the three indicators aforementioned, at a population level. These results suggest that HIV diagnosis remains a traumatic experience in the Sub-Saharan migrant population living in France, even if it does not have significant impact in terms of job or partner loss. These findings also bring new elements on the respective influence of migration and HIV diagnosis on the difficulties encountered by African migrants living with HIV, and on the so-called “normalization” of HIV.** | Manuscript p.15-16 |
| Limitations | 19 | Discuss limitations of the study, taking into account sources of potential bias or imprecision. Discuss both direction and magnitude of any potential bias  **(Discussion) Our study has some limitations. First, we used synthetic indicators for activity and union (such as to be in union or not, to be inactivity or not) and they cannot take into account changes in activity or partnership, thus we could not take into account to the fact that work conditions could deteriorate after diagnosis for example. Also, the impact of HIV diagnosis on union status could possibly be found in a difficulty to find new partner (celibacy) rather than losing a partner. Further research could investigate whether persons living with HIV take longer to find a partner because of their HIV status. Secondly, as the impact assessment as such is a methodological challenge, it may be especially difficult to assess impact retrospectively. Potential biases include the reconstruction of coherent meaning by the interviewees (32,33). However, the life-event method of data collection facilitates recall of events and their ordering in time and it substantially reduces this bias (34,35). The retrospective data collection of perceived well-being may suffer from a perception bias, as seen above.**  **Finally, it would have been interesting to take into account in our analyses a measure of persons’ health conditions at time of diagnosis, but it was not available in the survey.** | Manuscript p.18-19 |
| Interpretation | 20 | Give a cautious overall interpretation of results considering objectives, limitations, multiplicity of analyses, results from similar studies, and other relevant evidence  **(Conclusion) Migration entails great consequences for people’s lives. Public policy aiming at improving life with HIV should thus specifically address the difficult situation faced by Sub-Saharan migrants when they arrive in France. HIV diagnosis can trigger a degradation of well-being, but times are changing : with the generalization of effective ARTs, HIV diagnosis is less frightening that it used to be. Nevertheless, even is the situation is improving, hardship around diagnosis remains and further research is needed to understand why stigmatization remains important in the era of generalized antiretroviral therapy and how this can interact with persons’ well-being.** | Manuscript p.19 |
| Generalisability | 21 | Discuss the generalisability (external validity) of the study results  **The study was led in Paris area, but 60% of Sub-Saharan migrants in France live in this region.** | Manuscript p.19 |
| **Other information** |  |  |  |
| Funding | 22 | Give the source of funding and the role of the funders for the present study and, if applicable, for the original study on which the present article is based  ***Role of the funding source:***  ***The funders of the study had no role in the study design, data collection, data analysis, data interpretation, or writing of the paper. AG had full access to all the data and final responsibility for the decision to submit for publication.*** |  |

*Give information separately for exposed and unexposed groups.
