# Supplementary material for: "Times Are Changing": The Impact of HIV Diagnosis on Sub-Saharan Migrants’ Lives in France
Source: PLoS One. 2017 Jan 27;12(1):e0170226. doi: 10.1371/journal.pone.0170226 (PMC5271323; doi:10.1371/journal.pone.0170226)
Supplement: S1 Fig — (DOCX) [file pone.0170226.s004.docx]

*0.09*

*na*

*0.09*

*0.60*

*0.73*

*0.02*

*0.73*

*0.12*

*0.03*

*0.26*

**S1 Figure. Proportion of persons in activity, union and well-being the year before and the year after chronic Hepatitis B diagnosis, according to the period of diagnosis (N=258)**

*Scope : men (N=192) and women (N=66) from Hepatitis B group who have not yet migrated or being diagnosed at 18 years of age, who are observed at least five years after diagnosis who were diagnosed in France at least one year after arrival*

*Source : ANRS Parcours Survey 2012-2013*
